# Prevalence and attributable health burdens of vector-borne parasitic infectious diseases of poverty, 1990–2021: findings from the Global Burden of Disease Study 2021

**DOI:** 10.1186/s40249-024-01260-x

**Published:** 2024-12-11

**Authors:** Yin-Shan Zhu, Zhi-Shan Sun, Jin-Xin Zheng, Shun-Xian Zhang, Jing-Xian Yin, Han-Qing Zhao, Hai-Mo Shen, Gad Baneth, Jun-Hu Chen, Kokouvi Kassegne

**Affiliations:** 1 School of Global Health, Shanghai Jiao Tong University School of Medicine, Shanghai, 200025 China; 2 Longhua Hospital, Shanghai University of Traditional Chinese Medicine, Shanghai, China; 3 National Key Laboratory of Intelligent Tracking and Forecasting for Infectious Diseases, National Institute of Parasitic Diseases, Chinese Center for Disease Control and Prevention (Chinese Center for Tropical Diseases Research), National Health Commission of the People’s Republic of China (NHC) Key Laboratory of Parasite and Vector Biology, World Health Organization (WHO) Collaborating Center for Tropical Diseases, National Center for International Research On Tropical Diseases, Shanghai, 200025 China; 4 Koret School of Veterinary Medicine, The Hebrew University of Jerusalem, Rehovot, Israel; 5 Hainan Tropical Diseases Research Center (Hainan Sub-Center, Chinese Center for Tropical Diseases Research), Haikou, 571199 China

**Keywords:** Global burden of disease, Malaria, Leishmaniasis, Lymphatic filariasis, African trypanosomiasis, Chagas disease, Onchocerciasis, Sociodemographic index

## Abstract

**Background:**

Vector-borne parasitic infectious diseases associated with poverty (referred to as vb-pIDP), such as malaria, leishmaniasis, lymphatic filariasis, African trypanosomiasis, Chagas disease, and onchocerciasis, are highly prevalent in many regions around the world. This study aims to characterize the recent burdens of and changes in these vb-pIDP globally and provide a comprehensive and up-to-date analysis of geographical and temporal trends.

**Methods:**

Data on the prevalence and disability-adjusted life years (DALYs) of the vb-pIDP were retrieved from the Global Burden of Disease, Injuries, and Risk Factors Study (GBD) 2021 for 21 geographical regions and 204 countries worldwide, from 1990–2021. The age-standardized prevalence rate and DALYs rate by age, sex, and sociodemographic index (SDI) were calculated to quantify temporal trends. Correlation analysis was performed to examine the relationship between the age-standardized rate and the SDI.

**Results:**

Over the past 30 years, the age-standardized prevalence rate and DALYs rate of these vb-pIDP have generally decreased, with some fluctuations. The distribution of vb-pIDP globally is highly distinctive. Except for Chagas disease, the age-standardized prevalence rate and DALYs rate of other vb-pIDP were highest in low-SDI regions by 2021. Malaria had the highest age-standardized prevalence rate (2336.8 per 100,000 population, 95% UI: 2122.9, 2612.2 per 100,000 population) and age-standardized DALYs rate (806.0 per 100,000 population, 95% UI: 318.9, 1570.2 per 100,000 population) among these six vb-pIDP globally. Moreover, significant declines in the age-standardized prevalence rate and DALYs rate have been observed in association with an increase in the SDI . Globally, 0.14% of DALYs related to malaria are attributed to child underweight, and 0.08% of DALYs related to malaria are attributed to child stunting.

**Conclusions:**

The age-standardized prevalence rate and DALY rates for the vb-pIDP showed pronounced decreasing trends from 1990–2021. However, the vb-pIDP burden remains a substantial challenge for vector-borne infectious disease control globally and requires effective control strategies and healthcare systems. The findings provide scientific evidence for designing targeted health interventions and contribute to improving the prevention and control of infectious diseases.

**Graphical Abstract:**

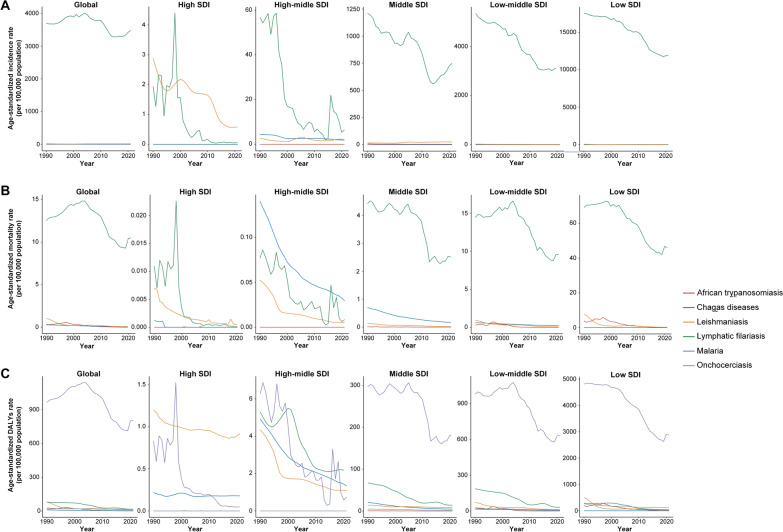

**Supplementary Information:**

The online version contains supplementary material available at 10.1186/s40249-024-01260-x.

## Background

Poverty-related infectious diseases are a group of diseases that are prevalent mainly in resource-poor areas [1]. Among them, vector-borne parasitic infectious diseases of poverty (hereafter referred to as vb-pIDP) are human illnesses caused by parasitic pathogens that are transmitted by vectors such as mosquitoes, ticks, sandflies, and other arthropods [2, 3]. Vb-pIDP in particular poses a significant burden on global health, especially in developing countries [4]. Malaria, leishmaniasis, lymphatic filariasis, African trypanosomiasis, Chagas disease, and onchocerciasis are major vb-pIDP that collectively cause more than one million deaths annually and afflict hundreds of millions more [5, 6]. Malaria is caused by *Plasmodium* parasites transmitted by *Anopheles* mosquitoes and causes an estimated 219 million cases and half a million deaths per year, mostly in children under five years of age in sub-Saharan Africa [7, 8]. Leishmaniasis is caused by *Leishmania* parasites and is transmitted by sandflies. Visceral leishmaniasis is the most severe form and is potentially fatal if untreated [2, 5]. Lymphatic filariasis, caused by filarial parasites such as *Wuchereria bancrofti*, is transmitted by mosquitoes such as *Anopheles*, *Culex*, and *Aedes*. Over 120 million people are affected, with 1.3 billion at risk in the tropics [9]. It causes elephantiasis and hydrocele. African trypanosomiasis is caused by *Trypanosoma* parasites transmitted by tsetse flies. It includes gambiense and rhodesiense forms, both of which cause sleeping sickness if left untreated [2, 5]. Chagas disease is caused by the parasite *Trypanosoma cruzi* and is transmitted by triatomine bug vectors [2, 5]. A major disease in Latin America that causes heart and intestinal complications. Onchocerciasis, caused by *Onchocerca volvulus* parasites, is transmitted by blackflies. It causes river blindness and severe skin disease, affecting more than 20 million people [5]. These tropical diseases are mostly neglected and are concentrated in impoverished communities lacking resources for prevention, diagnosis, and treatment. Integrated vector control, community mobilization, and new tools are crucial to combat their burden.

Although vb-pIDP generally does not present with acute onset, it can impair the health and productivity of animals and humans through widespread prevalence. Moreover, such diseases are widely distributed, ranging from deserts to rainforests. However, comprehensive estimates of the prevalence and subsequent impacts of the vb-pIDP remain scarce, leaving a gap for policymakers and service agencies dedicated to alleviating the burden of these diseases. In addition, the patterns cannot be discovered from the raw data in the database, so further analysis of these data can help relevant departments and individuals better understand the data, uncover hidden information, find value, support decision-making, optimize resource utilization, and improve predictive capabilities [10]. Considerable extensive literature on GBD research has clarified the research methods and detailed information regarding GBD 2021 [11–13]. In this study, data on the global burden of disease were utilized to describe the prevalence and disability-adjusted life years (DALYs) attributable to vb-pIDP at the global and regional levels across different sexes and age groups from 1990–2021 on the basis of the GBD 2021. This study excluded any personal or medical information of living individuals and did not involve animal experimentation. Moreover, it adheres to the Reporting of Studies Conducted via Observational Routinely Collected Health Data (RECORD) guidelines for research reporting.

## Methods

### Data sources

Data on the vb-pIDP were obtained from the Global Burden of Disease, Injuries, and Risk Factors Study (GBD) 2021 via the Global Health Data Exchange (GHDx) results tool (http://ghdx.healthdata.org/gbd-results-tool). The search criteria for the data used in this study in the GBD database are as follows: GBD estimate (causes of death or injury, risk factor), measure (DALYs, prevalence), metric (number, percentage, rate), cause (malaria, leishmaniasis, lymphatic filariasis, African trypanosomiasis, Chagas disease, onchocerciasis), location (global, custom regions), age (all ages, age-standardized), sex (both, male, female), and year range (1990–2021). Age was stratified into 17 groups: < 5, 5–9, 10–14, 15–19, 20–24, 25–29, 30–34, 35–39, 40–44, 45–49, 50–54, 55–59, 60–64, 65–69, 70–74, 75–79, and > 95 years. The GBD Study 2021 presented detailed and up-to-date estimates for 371 diseases and injuries and 88 risk factors for each of the 204 countries and territories, 21 regions, and both sexes and various age groups from 1990–2020 [14, 15], and the estimations of attributable burdens followed the general framework established for comparative risk assessment, which was described in a previous study [16]. Risk factors for malaria, including child underweight, child stunting, and child wasting, were extracted from the GBD 2021.

### Case definition

The study focused on six types of infectious diseases that were clearly diagnosed as specific vector-borne parasitic infectious diseases: malaria, leishmaniasis, lymphatic filariasis, African trypanosomiasis, Chagas disease, and onchocerciasis (Additional file 1: Table S1). For malaria, the International Classification of Diseases (ICD)−10 codes are B50–B50.0, B50.8–B52.0, B52.8–B53.1, B53.8–B54.0, and P37.3–P37.4, and the ICD-9 codes are 084–084.9, V12.03, and V75.1. For leishmaniasis, the ICD-10 codes are B55–B55.9, and the ICD-9 codes are 085–085.9, V05.2, and V75.2. For African trypanosomiasis, the ICD-10 codes are B56–B56.9, and the ICD-9 codes are 086.3–086.9, V75.3. For Chagas disease, the ICD-10 codes are B57–B57.5 and K93.1, and the ICD-9 codes are 086–086.2 and 425.5. For lymphatic filariasis, the ICD-10 codes are B74–B74.2, and the ICD-9 codes are 125.0–125.2. For onchocerciasis, the ICD-10 codes are B73–B73.1, and the ICD-9 codes are 125.3.

### Sociodemographic indices and geographic regions

The sociodemographic index (SDI) is a comprehensive assessment index that ranges from 0 (worst) to 100 (best). It is used to evaluate the level of development in the social, economic, and population health aspects of specific regions or countries. The index considers factors such as income level, educational attainment, and the fertility rate to compare social and economic development levels and population health status across different regions or countries. The SDI is calculated as the geometric mean of three factors: the total fertility rate for women under 25, the average years of schooling for individuals aged 15 and older, and the lag-distributed income index, which ranges from 0–1 [17]. In the 2021 GBD study, countries and regions were categorized into high, high-middle, middle, low-middle, or low-SDI regions on the basis of the quintiles of the SDI [15]. Furthermore, the study divided 204 countries and regions into 21 geographical regions (Additional file 1: Table S2). The global division of regions was based on similarities in epidemiology, disease distribution, causes of death, geographical factors, and other relevant considerations within each region.

### Statistical analysis

The prevalence and DALYs for these vb-pIDP cases were analysed for 1990 and 2021, both globally and regionally, to provide an overall impression of the burden. The ASRs of prevalence and DALYs across various factors, such as sex, age, year, and region, were then compared. This comparison included assessing variations between different levels of the SDI, regions, and countries. The comparison was made across 21 geographical regions and 204 countries and territories with different SDI levels. The ASRs (per 100,000 population) were extracted from the GBD database, and the formula was as follows:

$$\frac{{\mathop \sum \nolimits_{i = 1}^{N} \alpha_{i} W_{i} }}{{\mathop \sum \nolimits_{i = 1}^{N} W_{i} }}$$

In the ASR formula, ${\alpha }_{i}$ represents the age-specific rates for the ith age group. ${W}_{i}$ represents the number (or weight) of individuals in the same age group in the GBD standard population. *N* is the number of age groups. The 95% uncertainty interval (UI) is defined as the 2.5th and 97.5th values of an ordered draw of 1000 iterations.

The years of life lost (YLLs) for each vb-pIDP were calculated by multiplying the estimated deaths by the remaining life expectancy at the time of death. The years lived with disability (YLDs) were determined by multiplying disease incidence by disability weight. DALYs represent the total years lost due to disease, disability, or premature death, calculated as the sum of YLLs and YLDs [18–20]. Smoothing spline models were used to assess the relationships between different disease burden metrics, such as prevalence and DALYs, and the varying levels of the SDI across different vb-pIDP. This study uses the local weighted scatterplot smoothing (LOWESS) method to fit smooth splines, which automatically determines the order, number, and location of knots (nodes) on the basis of the data and the span parameter. The GBD database uses uncertainty intervals (UIs) rather than precise statistical values. Consequently, in this study, statistical significance cannot be directly calculated when comparing two numerical values (such as numbers and rates), only UIs are provided. If the UIs overlap, there is no significant difference (*P* > 0.05). Conversely, if the UIs do not overlap, a significant difference is suggested (*P* < 0.05). All data analysis and processing were performed via *R* software version 4.3.2 (R Foundation for Statistical Computing, Vienna, Austria).

## Results

### Prevalence and DALYs of the six notable vb-pIDP and temporal trends

In 2021, there were 173.9 million malaria cases (95% UI: 158.0, 194.5) worldwide, representing an increase of 9.0% compared with 1990, and the global age-standardized prevalence rate for malaria was 2336.8 per 100,000 population (95% UI: 2122.9, 2612.2 per 100,000 population), with a decrease of − 16.5% (95% UI: − 26.4, − 5.3%) compared with 1990, and there was no upwards or downwards trend (*P* > 0.05). The global age-standardized DALYs rate for malaria was estimated at 806.0 per 100,000 population (95% UI: 318.9, 1570.2 per 100,000 population), with a percentage change of −16.5% (95% UI: − 36.8, 2.5%), compared with 1990, and there was no significant upwards or downwards trend (*P* > 0.05) (Table 1). In the past 30 years, the age-standardized prevalence rate of malaria has declined in high-SDI regions (% change = − 77.1, 95% UI: − 86.5, − 61.0), high-middle-SDI regions (% change = − 81.2, 95% UI: − 90.6, − 65.6), middle-SDI regions (% change = − 36.4, 95% UI: − 57.0, − 5.0), low-middle-SDI regions (% change = − 41.7, 95% UI: − 52.8, − 29.5), and low-SDI regions (% change = − 44.9, 95% UI: − 53.4, − 35.6), and there has been a significant downwards trend across various SDI categories (*P* < 0.05). Malaria was most prevalent across the GBD central sub-Saharan Africa, at 21,328.8 per 100,000 population (95% UI: 16,092.3, 28,557.4 per 100,000 population), and 38,056.5 per 100,000 population (95% UI: 33,491.8, 42,691.8 per 100,000 population) in 1990. Andean Latin America, central Asia, East Asia, and southern Latin America had the largest declines (more than 80%) in malaria prevalence over the past 30 years. The age-standardized DALYs rate significantly declined in the high-SDI regions (% change = − 94.2, 95% UI: − 96.5, − 89.7), which showed a pronounced downwards trend (*P* < 0.05). However, the age-standardized DALYs rate for malaria remained relatively high in western sub-Saharan Africa, central sub-Saharan Africa, and eastern sub-Saharan Africa (Table 1).

**Table 1:** Age-standardized prevalence rate and DALYs rate for malaria in 1990 and 2021 and the percentage change in the age-standardized rates per 100,000 population by GBD region from 1990–2021

Malaria	Prevalence			DALYs		
	Age-standardized prevalence rate per 100,000 population (95% UI) 1990	Age-standardized prevalence rate per 100,000 population (95% UI) 2021	% change in age-standardized prevalence rate 1990–2021	Age-standardized DALYs rate per 100,000 population (95% UI) 1990	Age-standardized DALYs rate per 100,000 population (95% UI) 2021	% change in age-standardized DALYs rate 1990–2021
Global	2797.6 (2605.9, 3076.7)	2336.8 (2122.9, 2612.2)	− 16.5 (− 26.4, − 5.3)	965.7 (502.6, 1898.1)	806.0 (318.9, 1570.2)	− 16.5 (− 36.8, 2.5)
SDI groups						
High SDI	4.8 (3.8, 5.9)	1.1 (0.7, 1.7)	− 77.1 (− 86.5, − 61.0)	0.8 (0.5, 1.4)	0.05 (0.03, 0.07)	− 94.2 (− 96.5, − 89.7)
High-middle SDI	58.7 (39.3, 107.7)	11.0 (7.3, 17.0)	− 81.2 (− 90.6, − 65.6)	6.3 (1.0, 42.80)	0.7 (0.1, 1.9)	− 88.2 (− 95.4, − 71.2)
Middle SDI	869.1 (755.7, 1042.6)	552.3 (380.0, 789.5)	− 36.4 (− 57.0, − 5.0)	297.4 (134.7, 690.5)	177.6 (70.1, 334.7)	− 40.3 (− 56.3, − 23.8)
Low-middle SDI	3754.0 (3294.9, 4457.3)	2188.8 (1813.3, 2539.4)	− 41.7 (− 52.8, − 29.5)	978.8 (448.7, 2297.6)	630.9 (249.1, 1273.7)	− 35.5 (− 49.4, − 21.1)
Low-SDI	17,155.4 (15,844.1, 18,797.5)	9458.6 (8494.2, 10,473.8)	− 44.9 (− 53.4, − 35.6)	4811.5 (2580.7, 8569.1)	2869.2 (1107.4, 5683.9)	− 40.4 (− 57.6, − 27.6)
Geographic regions						
Andean Latin America	1333.0 (895.3, 2551.2)	241.0 (86.3, 311.5)	− 81.9 (− 92.8, − 70.9)	253.4 (57.0, 653.9)	3.3 (1.2, 11.1)	− 98.7 (− 99.1, − 97.5)
Australasia	0.0 (0.0, 0.0)	0.0 (0.0, 0.0)	0.0 (0.0, 0.0)	0.0 (0.0, 0.0)	0.0 (0.0, 0.0)	NA
Caribbean	187.1 (148.5, 297.7)	144.5 (82.3, 287.2)	− 22.8 (− 61.2, 59.4)	146.8 (54.7, 345.0)	103.7 (15.4, 321.9)	− 29.4 (− 70.3, − 4.9)
Central Asia	33.0 (12.7, 90.4)	0.0 (0.0, 0.0)	− 100.0 (− 100.0, − 100.0)	31.3 (4.8, 87.8)	0.0 (0.0, 0.0)	− 100.0 (− 100.0, − 100.0)
Central Europe	0.0 (0.0, 0.0)	0.0 (0.0, 0.0)	0.0 (0.0, 0.0)	0.0 (0.0, 0.0)	0.0 (0.0, 0.0)	NA
Central Latin America	472.4 (448.2, 493.8)	203.5 (156.7, 260.6)	− 56.9 (− 66.6, − 44.7)	42.6 (22.2, 79.2)	13.0 (3.0, 30.8)	− 69.5 (− 85.7, − 59.3)
Central sub-Saharan Africa	38,056.5 (33,491.8, 42,691.8)	21,328.8 (16,092.3, 28,557.4)	− 44.0 (− 59.2, − 24.8)	8226.0 (4631.1, 13,128.4)	4076.7 (1862.0, 7643.6)	− 50.4 (− 62.6, − 38.5)
East Asia	63.9 (36.0, 78.0)	2.4 (2.2, 2.7)	− 96.2 (− 97.0, − 93.2)	5.0 (0.3, 54.6)	0.02 (0.01, 0.02)	− 99.7 (− 100.0, − 93.8)
Eastern Europe	0.0 (0.0, 0.0)	0.0 (0.0, 0.0)	0.0 (0.0, 0.0)	0.0 (0.0, 0.0)	0.0 (0.0, 0.0)	NA
Eastern sub-Saharan Africa	18,142.2 (16,792.0, 19,958.63)	7386.3 (6348.7, 8354.0)	− 59.3 (− 65.1, − 53.1)	5089.4 (2906.4, 8550.2)	2199.7 (830.7, 4301.6)	− 56.8 (− 72.7, − 46.2)
High-income Asia Pacific	13.2 (9.0, 18.7)	6.6 (3.7, 11.0)	− 50.3 (− 74.3, − 6.5)	1.2 (1.0, 1.5)	0.03 (0.02, 0.04)	− 97.6 (− 98.2, − 96.8)
High-income North America	0.0 (0.0, 0.0)	0.0 (0.0, 0.0)	0.0 (0.0, 0.0)	0.0 (0.0, 0.0)	0.0 (0.0, 0.0)	NA
North Africa and Middle East	647.3 (474.7, 979.7)	450.3 (308.2, 674.1)	− 30.4 (− 61.5, 24.6)	195.7 (74.2, 460.1)	125.5 (37.8, 272.1)	− 35.8 (− 53.1, − 15.5)
Oceania	12,770.8 (5653.7, 35,627.7)	5622.4 (5047.0, 6243.0)	− 56.0 (− 84.5, − 1.1)	2624.3 (580.9, 8023.6)	1047.5 (474.8, 1931.0)	− 60.1 (− 75.1, − 22.9)
South Asia	1453.3 (1007.4, 2430.5)	321.3 (264.8, 419.9)	− 77.9 (− 88.3, − 66.1)	597.8 (226.8, 1948.6)	104.1 (6.9, 344.8)	− 82.6 (− 97.0, − 73.5)
Southeast Asia	918.4 (750.5, 1229.0)	248.3 (176.0, 284.1)	− 73.0 (− 81.6, − 64.9)	177.0 (49.3, 710.4)	16.1 (5.2, 52.1)	− 90.9 (− 92.9, − 82.3)
Southern Latin America	45.4 (44.8, 46.1)	0.0 (0.0, 0.0)	− 100.0 (− 100.0, − 100.0)	3.2 (2.5, 4.0)	0.0 (0.0, 0.0)	− 100.0 (− 100.0, − 100.0)
Southern sub-Saharan Africa	534.7 (380.3, 887.5)	239.7 (182.7, 311.5)	− 55.2 (− 74.9, − 30.5)	375.9 (136.1, 823.2)	179.6 (77.7, 376.8)	− 52.2 (− 65.0, − 23.5)
Tropical Latin America	484.7 (418.9, 560.8)	126.6 (92.7, 168.1)	− 73.9 (− 81.7, − 63.9)	171.3 (63.9, 339.4)	6.0 (1.5, 15.6)	− 96.5 (− 98.2, − 95.4)
Western Europe	0.0 (0.0, 0.0)	0.0 (0.0, 0.0)	0.0 (0.0, 0.0)	0.11 (0.06, 0.17)	0.0 (0.0, 0.0)	− 100.0 (− 100.0, − 100.0)
Western sub-Saharan Africa	33,437.3 (30,122.5, 37,563.5)	17,793.1 (14,749.2, 20,773.1)	− 46.8 (− 56.7, − 36.3)	8231.7 (4281.6, 14,191.0)	5668.4 (2216.2, 11,127.5)	− 31.1 (− 48.0, − 17.8)

*DALYs* disability-adjusted life years, *GBD* global burden of disease, *SDI* sociodemographic index, *UI* uncertainty interval, *NA* not applicable

In 2021, there were 6.2 million cases of leishmaniasis worldwide (95% UI: 5.8, 6.7), representing an increase of 95.0% from 1990, and the global age-standardized prevalence rate was 77.0 per 100,000 population (95% UI: 71.9, 83.0 per 100,000 population), up by 22.3% (95% UI: 5.8, 40.5%) from 1990, with no significant upwards or downwards trend (*P* > 0.05). The global age-standardized DALYs rate was 10.3 per 100,000 population (95% UI: 6.0, 22.0 per 100,000 population), with a percentage change of −87.1% (95% UI: −91.7, −76.7%), compared with 1990, with a significant downwards trend (*P* < 0.05) (Table 2). Over the past 30 years, the age-standardized prevalence rate has declined in high-middle-SDI regions (% change = − 0.4%, 95% UI: − 30.6, 53.8) and low-middle-SDI regions (% change = − 26.8, 95% UI: − 39.9, − 12.4), and there has been no upwards or downwards trend, *P* > 0.05. However, it increased in the high-SDI regions (% change = 18.1, 95% UI: − 21.2, 129.3), middle-SDI regions (% change = 26.6, 95% UI: − 1.0, 59.4), and low-SDI regions (% change = 42.0, 95% UI: 1.6, 97.5). Leishmaniasis was most prevalent across GBD North Africa and the Middle East region, with a rate of 642.9 per 100,000 population (95% UI: 592.1, 712.9 per 100,000 population) in 2021, and it slightly decreased from 645.7 per 100,000 population (95% UI: 480.8, 883.7 per 100,000 population) in 1990. The age-standardized DALYs rate decreased in the high-SDI regions (% change = − 23.5, 95% UI: − 75.2, 99.0), high-middle-SDI regions (% change = − 74.6, 95% UI: − 90.7, 25.0), middle-SDI regions (% change = − 40.5, 95% UI: − 75.4, 47.7), low-middle-SDI regions (% change = − 83.3, 95% UI: − 89.3, − 61.0), and low-SDI regions (% change = − 95.0, 95% UI: − 96.8, − 91.3), and there was a significant downwards trend in the low-SDI regions (*P* < 0.05). However, the age-standardized DALYs rate for leishmaniasis was higher in tropical Latin America, North Africa, the Middle East, and Andean Latin America than in other regions (Table 2).

**Table 2:** Age-standardized prevalence rate and DALYs rate for leishmaniasis in 1990 and 2021 and the percentage change in the age-standardized rates per 100,000 population by GBD region from 1990–2021

Leishmaniasis	Prevalence			DALYs		
	Age-standardized prevalence rate per 100,000 population (95% UI) 1990	Age-standardized prevalence rate per 100,000 population (95% UI) 2021	% change in age-standardized prevalence rate 1990–2021	Age-standardized DALYs rate per 100,000 population (95% UI) 1990	Age-standardized DALYs rate per 100,000 population (95% UI) 2021	% change in age-standardized DALYs rate 1990–2021
Global	62.9 (52.9, 76.6)	77.0 (71.9, 83.0)	22.3 (5.8, 40.5)	79.6 (29.9, 246.6)	10.3 (6.0, 22.0)	− 87.1 (− 91.7, − 76.7)
SDI groups						
High SDI	11.8 (5.0, 23.5)	13.9 (10.4, 19.3)	18.1 (− 21.2, 129.3)	1.2 (0.3, 4.9)	0.9 (0.6, 1.4)	− 23.5 (− 75.2, 99.0)
High-middle SDI	12.0 (6.9, 21.1)	11.9 (10.0, 14.9)	− 0.4 (− 30.6, 53.8)	4.3 (0.5, 32.7)	1.1 (0.656, 32.)	− 74.6 (− 90.7, 25.0)
Middle SDI	84.0 (63.3, 117.0)	106.4 (99.0, 117.2)	26.6 (− 1.0, 59.4)	14.0 (3.7, 58.4)	8.3 (4.9, 14.5)	− 40.5 (− 75.4, 47.7)
Low-middle SDI	136.2 (102.6, 192.5)	99.7 (88.7, 115.9)	− 26.8 (− 39.9, − 12.4)	73.2 (18.5, 276.5)	12.2 (6.0, 32.1)	− 83.3 (− 0.893, − 61.0)
Low-SDI	98.3 (66.3, 146.4)	139.7 (125.5, 156.6)	42.0 (1.6, 97.5)	504.9 (200.6, 1383.7)	25.2 (15.1, 47.5)	− 95.0 (− 96.8, − 91.3)
Geographic regions						
Andean Latin America	551.9 (389.4, 778.5)	548.7 (487.3, 619.8)	− 0.6 (− 22.7, 30.8)	47.2 (22.4, 98.0)	35.5 (23.8, 50.6)	− 24.9 (− 62.8, 22.7)
Australasia	0.0 (0.0, 0.0)	0.0 (0.0, 0.0)	0.0 (0.0, 0.0)	0.0 (0.0, 0.0)	0.0 (0.0, 0.0)	NA
Caribbean	55.3 (30.0, 97.6)	52.9 (43.2, 66.2)	− 4.3 (− 31.5, 51.0)	3.8 (1.9, 7.0)	3.4 (2.2, 4.9)	− 11.6 (− 38.4, 44.1)
Central Asia	218.9 (168.6, 288.8)	111.9 (93.5, 135.5)	− 48.9 (− 54.1, − 41.7)	15.5 (8.8, 28.1)	9.4 (4.8, 28.3)	− 39.1 (− 55.0, 0.6)
Central Europe	1.4 (1.0, 1.9)	0.7 (0.6, 0.8)	− 50.6 (− 61.1, − 35.8)	8.5 (0.1, 80.6)	0.4 (0.0, 3.8)	− 94.9 (− 95.7, − 46.6)
Central Latin America	241.1 (170.5, 351.9)	215.9 (193.2, 249.7)	− 10.5 (− 29.5, 16.1)	16.9 (9.5, 29.1)	14.1 (9.3, 20.5)	− 16.2 (− 39.7, 13.8)
Central sub-Saharan Africa	27.3 (13.5, 50.1)	16.2 (12.3, 22.3)	− 40.7 (− 61.0, 7.1)	389.4 (205.1, 617.2)	26.8 (14.0, 43.1)	− 93.1 (− 94.3, − 91.8)
East Asia	2.4 (1.1, 5.7)	1.1 (0.6, 2.2)	− 56.0 (− 63.7, − 38.1)	5.2 (0.1, 28.2)	0.8 (0.0, 4.4)	− 84.3 (− 87.1, − 49.5)
Eastern Europe	0.0 (0.0, 0.0)	0.0 (0.0, 0.0)	0.0 (0.0, 0.0)	0.0 (0.0, 0.0)	0.0 (0.0, 0.0)	NA
Eastern sub-Saharan Africa	25.8 (17.1, 36.8)	11.0 (9.2, 13.6)	− 57.3 (− 67.1, − 42.9)	653.3 (442.5, 909.0)	23.0 (13.9, 34.6)	− 96.5 (− 97.4, − 95.5)
High-income Asia Pacific	0.0 (0.0, 0.0)	0.0 (0.0, 0.0)	0.0 (0.0, 0.0)	0.0 (0.0, 0.0)	0.0 (0.0, 0.0)	NA
High-income North America	0.1 (0.0, 0.2)	0.10 (0.06, 0.16)	10.7 (− 41.5, 255.0)	0.01 (0.00, 0.02)	0.01 (0.00, 0.01)	10.9 (− 41.5, 255.0)
North Africa and Middle East	645.7 (480.8, 883.7)	642.9 (592.1, 712.9)	− 0.4 (− 20.3, 26.4)	96.4 (27.8, 519.2)	45.2 (28.4, 79.8)	− 53.1 (− 84.6, 20.4)
Oceania	0.0 (0.0, 0.0)	0.0 (0.0, 0.0)	0.0 (0.0, 0.0)	0.0 (0.0, 0.0)	0.0 (0.0, 0.0)	NA
South Asia	23.8 (15.2, 35.4)	18.9 (16.2, 22.6)	− 20.7 (− 41.2, 12.2)	153.0 (1.4, 693.4)	5.9 (0.9, 26.1)	− 96.2 (− 97.1, − 18.6)
Southeast Asia	1.1 (0.6, 2.0)	2.1 (1.8, 2.4)	91.1 (17.1, 250.8)	0.2 (0.0, 0.5)	0.15 (0.09, 0.22)	− 12.9 (− 64.1, 193.7)
Southern Latin America	13.0 (5.2, 28.6)	12.9 (10.3, 17.5)	− 1.3 (− 39.9, 106.3)	1.2 (0.3, 4.7)	0.9 (0.5, 1.8)	− 24.0 (− 60.6, 109.2)
Southern sub-Saharan Africa	0.7 (0.3, 1.5)	0.7 (0.5, 0.9)	− 9.9 (− 42.2, 73.1)	0.05 (0.02, 0.10)	0.04 (0.03, 0.06)	− 10.4 (− 44.8, 76.4)
Tropical Latin America	302.6 (234.5, 390.3)	297.5 (269.8, 330.7)	− 1.7 (− 18.2, 19.9)	64.4 (14.3, 191.4)	52.5 (14.8, 143.6)	− 18.5 (− 30.5, 14.3)
Western Europe	1.0 (1.0, 1.7)	0.8 (0.6, 1.0)	− 23.8 (− 41.4, 6.9)	1.6 (0.4, 10.8)	0.5 (0.2, 2.7)	− 68.8 (− 75.1, − 48.5)
Western sub-Saharan Africa	35.7 (22.1, 55.1)	41.9 (36.6, 48.4)	17.5 (− 14.4, 72.4)	2.9 (1.8, 4.6)	5.5 (3.7, 7.8)	88.3 (32.0, 169.8)

*DALYs* disability-adjusted life years, *GBD* global burden of disease, *SDI* sociodemographic index, *UI* uncertainty interval, *NA* not applicable

For lymphatic filariasis, there were 56.9 million cases (95% UI: 48.7, 67.9) globally, representing a decrease of 72.8% compared with 1990, and the estimated global age-standardized prevalence rate was estimated at 706.0 per 100,000 population (95% UI: 603.7, 841.8 per 100,000 population) in 2021, with a decrease of − 82.6% (95% UI: − 85.8, − 78.7%) compared with 1990. In 2021, the global age-standardized DALYs rate was estimated at 16.5 per 100,000 population (95% UI: 9.7, 28.0 per 100,000 population), which was a decrease of − 79.1% (95% UI: − 84.2, − 70.5%) compared with that in 1990 (Table 3). Lymphatic filariasis was most prevalent across GBD Oceania, with 7646.7 per 100,000 population (95% UI: 2970.4, 18,140.4 per 100,000 population) in 2021 and a decrease from 33,028.4 per 100,000 population (95% UI: 17,334.7, 52,695.9 per 100,000 population) in 1990. Oceania and southern sub-Saharan Africa also experienced declines in the prevalence of malaria. Additionally, over the past 30 years, the age-standardized prevalence rate of lymphatic filariasis decreased in the high-middle-SDI regions (% change = − 70.7, 95% UI: − 90.0, − 15.2), middle-SDI regions (% change = − 82.7, 95% UI: − 89.4, − 72.3), low-middle-SDI regions (% change = − 85.9, 95% UI: − 88.6, − 82.6), and low-SDI regions (% change = − 88.4, 95% UI: − 90.9, − 85.7), and the age-standardized DALYs rate also decreased in the high-middle-SDI regions (% change = − 59.3, 95% UI: − 68.8, − 44.2), middle-SDI regions (% change = − 79.5, 95% UI: − 84.6, − 70.7), low-middle-SDI regions (% change = − 83.9, 95% UI: − 87.8, − 77.8), and low-SDI regions (% change = − 86.6, 95% UI: − 90.1, − 80.8). However, the age-standardized DALYs rate for lymphatic filariasis was higher in Oceania, western sub-Saharan Africa, South Asia, and central sub-Saharan Africa (Table 3).

**Table 3:** Age-standardized prevalence rate and DALYs rate for lymphatic filariasis in 1990 and 2021 and the percentage change in the age-standardized rates per 100,000 population by GBD region from 1990–2021

Lymphatic filariasis	Prevalence			DALYs		
	Age-standardized prevalence rate per 100,000 population (95% UI) 1990	Age-standardized prevalence rate per 100,000 population (95% UI) 2021	% change in age-standardized prevalence rate 1990–2021	Age-standardized DALYs rate per 100,000 population (95% UI) 1990	Age-standardized DALYs rate per 100,000 population (95% UI) 2021	% change in age-standardized DALYs rate 1990–2021
Global	4055.0 (3493.5, 4829.1)	706.0 (603.7, 841.8)	− 82.6 (− 85.8, − 78.7)	79.1 (53.5, 110.5)	16.5 (9.7, 28.0)	− 79.1 (− 84.2, − 70.5)
SDI groups						
High SDI	0.0 (0.0, 0.0)	0.0 (0.0, 0.0)	0.0 (0.0, 0.0)	0.0 (0.0, 0.0)	0.0 (0.0, 0.0)	NA
High-middle SDI	256.9 (121.6, 572.2)	75.3 (37.8, 177.6)	− 70.7 (− 90.0, − 15.2)	5.3 (3.4, 7.8)	2.2 (1.2, 3.8)	− 59.3 (− 68.8, − 44.2)
Middle SDI	3346.2 (2336.3, 5033.8)	580.0 (461.0, 826.5)	− 82.7 (− 89.4, − 72.3)	67.3 (45.4, 94.9)	13.8 (7.9, 23.8)	− 79.5 (− 84.6, − 70.7)
Low-middle SDI	9744.7 (8575.5, 11,330.6)	1370.2 (1150.1, 1664.5)	− 85.9 (− 88.6, − 82.6)	186.2 (127.2, 261.6)	30.0 (17.7, 50.3)	− 83.9 (− 87.8, − 77.8)
Low-SDI	14,389.4 (12,098.0, 17,028.6)	1669.8 (1371.0, 2093.6)	− 88.4 (− 90.9, − 85.7)	279.1 (189.9, 389.9)	37.4 (22.2, 62.0)	− 86.6 (− 90.1, − 80.8)
Geographic regions						
Andean Latin America	0.0 (0.0, 0.0)	0.0 (0.0, 0.0)	0.0 (0.0, 0.0)	0.0 (0.0, 0.0)	0.0 (0.0, 0.0)	NA
Australasia	0.0 (0.0, 0.0)	0.0 (0.0, 0.0)	0.0 (0.0, 0.0)	0.0 (0.0, 0.0)	0.0 (0.0, 0.0)	NA
Caribbean	6085.0 (2216.3, 13,582.0)	851.8 (465.2, 1642.7)	− 86.0 (− 95.0, − 54.9)	108.2 (73.7, 151.0)	21.2 (13.3, 34.9)	− 80.4 (− 84.6, − 73.1)
Central Asia	0.0 (0.0, 0.0)	0.0 (0.0, 0.0)	0.0 (0.0, 0.0)	0.0 (0.0, 0.0)	0.0 (0.0, 0.0)	NA
Central Europe	0.0 (0.0, 0.0)	0.0 (0.0, 0.0)	0.0 (0.0, 0.0)	0.0 (0.0, 0.0)	0.0 (0.0, 0.0)	NA
Central Latin America	0.0 (0.0, 0.0)	0.0 (0.0, 0.0)	0.0 (0.0, 0.0)	0.0 (0.0, 0.0)	0.0 (0.0, 0.0)	NA
Central sub-Saharan Africa	14,837.0 (8930.3, 23,403.9)	1989.9 (1060.3, 3782.6)	− 86.6 (− 93.4, − 73.3)	286.3 (192.4, 395.9)	39.1 (22.2, 67.6)	− 86.3 (− 90.6, − 79.4)
East Asia	0.0 (0.0, 0.0)	0.0 (0.0, 0.0)	0.0 (0.0, 0.0)	0.0 (0.0, 0.0)	0.0 (0.0, 0.0)	NA
Eastern Europe	0.0 (0.0, 0.0)	0.0 (0.0, 0.0)	0.0 (0.0, 0.0)	0.0 (0.0, 0.0)	0.0 (0.0, 0.0)	NA
Eastern sub-Saharan Africa	13,761.2 (9874.5, 18,730.8)	702.6 (470.0, 1088.8)	− 94.9 (− 96.9, − 91.7)	263.1 (178.9, 366.9)	24.6 (14.4, 43.7)	− 90.7 (− 93.3, − 85.6)
High-income Asia Pacific	2.3 (0.4, 17.0)	2.8 (0.7, 14.6)	18.7 (− 93.1, 1799.2)	0.06 (0.03, 0.10)	0.10 (0.05, 0.17)	70.8 (56.3, 85.8)
High-income North America	0.0 (0.0, 0.0)	0.0 (0.0, 0.0)	0.0 (0.0, 0.0)	0.0 (0.0, 0.0)	0.0 (0.0, 0.0)	NA
North Africa and Middle East	853.8 (233.7, 2755.1)	170.4 (48.6, 537.5)	− 80.0 (− 95.4, − 4.7)	15.6 (9.0, 25.6)	3.2 (1.9, 5.8)	− 79.5 (− 82.1, − 76.6)
Oceania	33,028.4 (17,334.7, 52,695.9)	7646.5 (2970.4, 18,140.4)	− 76.8 (− 92.0, − 35.3)	607.5 (419.6, 865.2)	163.5 (107.5, 237.3)	− 73.1 (− 79.0, − 66.8)
South Asia	11,084.2 (10,336.0, 11,905.2)	1884.3 (1646.1, 2191.4)	− 83.0 (− 84.2, − 81.4)	215.5 (147.3, 302.4)	39.6 (23.1, 66.4)	− 81.6 (− 86.3, − 74.6)
Southeast Asia	11,094.4 (5972.5, 18,901.6)	1089.1 (539.7, 2167.2)	− 90.2 (− 95.9, − 76.3)	225.4 (150.9, 315.8)	27.8 (16.0, 48.6)	− 87.7 (− 91.0, − 81.4)
Southern Latin America	0.0 (0.0, 0.0)	0.0 (0.0, 0.0)	0.0 (0.0, 0.0)	0.0 (0.0, 0.0)	0.0 (0.0, 0.0)	NA
Southern sub-Saharan Africa	300.5 (78.0, 1111.2)	150.9 (52.4, 490.5)	− 49.8 (− 90.5, 151.7)	6.4 (3.7, 11.2)	5.1 (2.9, 8.9)	− 21.1 (− 27.2, − 14.5)
Tropical Latin America	185.3 (132.7, 350.3)	7.9 (6.8, 13.6)	− 95.7 (− 97.9, − 92.8)	4.6 (3.0, 6.9)	0.7 (0.5, 1.0)	− 85.1 (− 88.7, − 81.1)
Western Europe	0.0 (0.0, 0.0)	0.0 (0.0, 0.0)	0.0 (0.0, 0.0)	0.0 (0.0, 0.0)	0.0 (0.0, 0.0)	NA
Western sub-Saharan Africa	17,366.9 (11,288.5, 25,504.1)	1729.1 (1105.9, 2662.4)	− 90.0 (− 94.4, − 82.3)	322.7 (219.1, 446.8)	41.1 (25.1, 66.6)	− 87.3 (− 90.4, − 82.3)

*DALYs* disability-adjusted life years, *GBD* global burden of disease, *SDI* sociodemographic index, *UI* uncertainty interval, *NA* not applicable

For African trypanosomiasis, there were 2367.9 cases (95% UI: 1130.4, 4391.7) globally, representing a decrease of 96.2% compared with 1990, and the global age-standardized prevalence rate was estimated at 0.03 per 100,000 population (95% UI: 0.01, 0.06 per 100,000 population) in 2021, decreasing by − 97.4% (95% UI: − 97.7, − 97.2%) compared with 1990, and there was a significant downwards trend (*P* < 0.05). The global age-standardized DALYs rate was 0.8 per 100,000 population (95% UI: 0.3, 2.3 per 100,000 population), with a percentage change of − 96.0% (95% UI: − 97.8, − 89.8%) compared with that in 1990, and there was a significant downwards trend (*P* < 0.05) (Table 4). From 1990–2021, the age-standardized prevalence rate of African trypanosomiasis declined in middle-SDI regions (% change = − 80.6, 95% UI: − 83.0, − 77.3), low-middle-SDI regions (% change = − 97.3, 95% UI: − 97.6, − 96.9), and low-SDI regions (% change = − 98.7, 95% UI: − 98.9, − 98.5), and there was a significant downwards trend in these regions (*P* < 0.05). The age-standardized DALYs rate also declined in the middle-SDI regions (% change = − 84.1, 95% UI: − 90.7, − 73.9), low-middle-SDI regions (% change = − 96.2, 95% UI: − 97.7, − 92.2), and low-SDI regions (% change = − 97.9, 95% UI: − 99.0, − 93.6), and there was a significant downwards trend in these regions, (*P* < 0.05). Notably, owing to the low prevalence of African trypanosomiasis in high-SDI regions, high-middle-SDI regions and some countries or insufficient research focus, there was a lack of relevant data in the GBD database used, which prevented accurate estimation (Table 4).

**Table 4:** Age-standardized prevalence rates and DALY rates for African trypanosomiasis in 1990 and 2021 and the percentage change in the age-standardized rates per 100,000 population by GBD region from 1990–2021

African trypanosomiasis	Prevalence			DALY		
	Age-standardized prevalence rate per 100,000 population (95% UI) 1990	Age-standardized prevalence rate per 100,000 population (95% UI) 2021	% change in Age-standardized prevalence rate 1990–2021	Age-standardized DALY rate per 100,000 population (95% UI) 1990	Age-standardized DALY rate per 100,000 population (95% UI) 2021	% change in age-standardized DALY rate 1990–2021
Global	1.2 (0.5, 2.2)	0.03 (0.01, 0.06)	− 97.4 (− 97.7, − 97.2)	19.9 (10.0, 31.9)	0.8 (0.3, 2.3)	− 96.0 (− 97.8, − 89.8)
SDI groups						
High SDI	0.0 (0.0, 0.0)	0.0 (0.0, 0.0)	0.0 (0.0, 0.0)	0.0 (0.0, 0.0)	0.0 (0.0, 0.0)	NA
High-middle SDI	0.0 (0.0, 0.0)	0.0 (0.0, 0.0)	0.0 (0.0, 0.0)	0.0 (0.0, 0.0)	0.0 (0.0, 0.0)	NA
Middle SDI	0.04 (0.02, 0.08)	0.01 (0.00, 0.02)	− 80.6 (− 83.0, − 77.3)	1.0 (0.4, 1.8)	0.2 (0.1, 0.3)	− 84.1 (− 90.7, − 73.9)
Low-middle SDI	1.3 (0.6, 2.5)	0.04 (0.02, 0.06)	− 97.3 (− 97.6, − 96.9)	17.1 (8.4, 28.2)	0.7 (0.3, 1.5)	− 96.2 (− 97.7, − 92.2)
Low-SDI	11.4 (5.1, 22.3)	0.2 (0.1, 0.3)	− 98.7 (− 98.9, − 98.5)	193.9 (94.4, 316.2)	4.1 (1.3, 13.0)	− 97.9 (− 99.0, − 93.6)
Geographic regions						
Andean Latin America	0.0 (0.0, 0.0)	0.0 (0.0, 0.0)	0.0 (0.0, 0.0)	0.0 (0.0, 0.0)	0.0 (0.0, 0.0)	NA
Australasia	0.0 (0.0, 0.0)	0.0 (0.0, 0.0)	0.0 (0.0, 0.0)	0.0 (0.0, 0.0)	0.0 (0.0, 0.0)	NA
Caribbean	0.0 (0.0, 0.0)	0.0 (0.0, 0.0)	0.0 (0.0, 0.0)	0.0 (0.0, 0.0)	0.0 (0.0, 0.0)	NA
Central Asia	0.0 (0.0, 0.0)	0.0 (0.0, 0.0)	0.0 (0.0, 0.0)	0.0 (0.0, 0.0)	0.0 (0.0, 0.0)	NA
Central Europe	0.0 (0.0, 0.0)	0.0 (0.0, 0.0)	0.0 (0.0, 0.0)	0.0 (0.0, 0.0)	0.0 (0.0, 0.0)	NA
Central Latin America	0.0 (0.0, 0.0)	0.0 (0.0, 0.0)	0.0 (0.0, 0.0)	0.0 (0.0, 0.0)	0.0 (0.0, 0.0)	NA
Central sub-Saharan Africa	94.4 (41.7, 183.2)	0.9 (0.5, 1.6)	− 99.0 (− 99.2, − 98.9)	1181.1 (585.3, 1964.8)	15.0 (7.4, 25.9)	− 98.7 (− 99.1, − 98.2)
East Asia	0.0 (0.0, 0.0)	0.0 (0.0, 0.0)	0.0 (0.0, 0.0)	0.0 (0.0, 0.0)	0.0 (0.0, 0.0)	NA
Eastern Europe	0.0 (0.0, 0.0)	0.0 (0.0, 0.0)	0.0 (0.0, 0.0)	0.0 (0.0, 0.0)	0.0 (0.0, 0.0)	NA
Eastern sub-Saharan Africa	9.7 (4.60, 18.0)	0.11 (0.07, 0.18)	− 98.8 (− 99.0, − 98.5)	261.8 (111.4, 466.6)	5.8 (0.8, 28.9)	− 97.8 (− 99.6, − 89.8)
High-income Asia Pacific	0.0 (0.0, 0.0)	0.0 (0.0, 0.0)	0.0 (0.0, 0.0)	0.0 (0.0, 0.0)	0.0 (0.0, 0.0)	NA
High-income North America	0.0 (0.0, 0.0)	0.0 (0.0, 0.0)	0.0 (0.0, 0.0)	0.0 (0.0, 0.0)	0.0 (0.0, 0.0)	NA
North Africa and Middle East	0.0 (0.0, 0.0)	0.0 (0.0, 0.0)	0.0 (0.0, 0.0)	0.0 (0.0, 0.0)	0.0 (0.0, 0.0)	NA
Oceania	0.0 (0.0, 0.0)	0.0 (0.0, 0.0)	0.0 (0.0, 0.0)	0.0 (0.0, 0.0)	0.0 (0.0, 0.0)	NA
South Asia	0.0 (0.0, 0.0)	0.0 (0.0, 0.0)	0.0 (0.0, 0.0)	0.0 (0.0, 0.0)	0.0 (0.0, 0.0)	NA
Southeast Asia	0.0 (0.0, 0.0)	0.0 (0.0, 0.0)	0.0 (0.0, 0.0)	0.0 (0.0, 0.0)	0.0 (0.0, 0.0)	NA
Southern Latin America	0.0 (0.0, 0.0)	0.0 (0.0, 0.0)	0.0 (0.0, 0.0)	0.0 (0.0, 0.0)	0.0 (0.0, 0.0)	NA
Southern sub-Saharan Africa	0.0 (0.0, 0.0)	0.0 (0.0, 0.0)	0.0 (0.0, 0.0)	0.0 (0.0, 0.0)	0.0 (0.0, 0.0)	NA
Tropical Latin America	0.0 (0.0, 0.0)	0.0 (0.0, 0.0)	0.0 (0.0, 0.0)	0.0 (0.0, 0.0)	0.0 (0.0, 0.0)	NA
Western Europe	0.0 (0.0, 0.0)	0.0 (0.0, 0.0)	0.0 (0.0, 0.0)	0.0 (0.0, 0.0)	0.0 (0.0, 0.0)	NA
Western sub-Saharan Africa	3.7 (1.4, 7.6)	0.2 (0.1, 0.5)	− 93.7 (− 94.3, − 93.2)	45.4 (18.7, 84.7)	4.1 (1.5, 8.4)	− 91.0 (− 95.1, − 85.1)

*DALYs* disability-adjusted life years, *GBD* global burden of disease, *SDI* sociodemographic index, *UI* uncertainty interval, *NA* not applicable

In 2021, 6.3 million people (95% UI: 5.4, 7.2) worldwide had Chagas disease, representing a decrease of 10.9% compared with 1990, and the estimated global age-standardized prevalence rate of Chagas disease in 2021 was 75.6 per 100,000 population (95% UI: 65.2, 86.5 per 100,000 population), with a percentage change of − 47.9% (95% UI: − 49.3, − 46.4%) compared with 1990, and there was a pronounced downwards trend (*P* < 0.05). Chagas disease was most prevalent across the GBD Southern Latin America, at 1346.1 per 100,000 population (95% UI: 1176.1, 1538.4 per 100,000 population) in 2021, with a decrease from 3944.8 per 100,000 population (95% UI: 3466.5, 4402.7 per 100,000 population) in 1990, followed by Andean Latin America, tropical Latin America, and Central Latin America, with a certain distribution pattern (Table 5). In 2021, the estimated global age-standardized DALYs rate was 2.8 per 100,000 population (95% UI: 2.4, 3.2 per 100,000 population), with a decline of −71.2% (95% UI: − 74.7, − 67.0%) compared with 1990, and there was a significant decrease (*P* < 0.05). Over the past 30 years, the age-standardized incidence rate of Chagas disease has declined in high-middle-SDI regions (% change = − 60.3, 95% UI: − 62.5, − 58.1), middle-SDI regions (% change = − 46.6, 95% UI: − 48.7, − 44.1), and low-middle-SDI regions (% change = − 48.1, 95% UI: − 50.4, − 46.0), and there has been a pronounced downwards trend in those regions (*P* < 0.05). Andean Latin America, Australasia, the Caribbean, central Latin America, southern Latin America, and tropical Latin America experienced decreases in the prevalence of Chagas disease. The age-standardized DALYs rate decreased in the high-SDI regions (% change = − 17.7, 95% UI: − 25.1, − 11.0), high-middle-SDI regions (% change = − 72.8, 95% UI: − 77.8, − 66.0), middle-SDI regions (% change = − 77.8, 95% UI: − 80.6, − 74.2), and low-middle-SDI regions (% change = − 64.7, 95% UI: − 71.1, − 56.1), and there were pronounced downwards trends in the high-middle-SDI regions, middle-SDI regions, and low-middle-SDI regions (*P* < 0.05) (Table 5).

**Table 5:** Age-standardized prevalence rates and DALY rates for Chagas disease in 1990 and 2021 and the percentage change in the age-standardized rates per 100,000 population by GBD region from 1990–2020

Chagas disease	Prevalence			DALY		
	Age-standardized prevalence rate per 100,000 population (95% UI) 1990	Age-standardized prevalence rate per 100,000 population (95% UI) 2021	% change in Age-standardized prevalence rate 1990–2021	Age-standardized DALY rate per 100,000 population (95% UI) 1990	Age-standardized DALY rate per 100,000 population (95% UI) 2021	% change in age-standardized DALY rate 1990–2021
Global	145.2 (125.4, 168.3)	75.6 (65.2, 86.5)	− 47.9 (− 49.3, − 46.4)	9.7 (8.5, 10.7)	2.8 (2.4, 3.2)	− 71.2 (− 74.7, − 67.0)
SDI groups						
High SDI	19.1 (16.0, 22.6)	19.5 (16.2, 22.9)	2.3 (− 2.1, 7.5)	0.22 (0.15, 0.32)	0.2 (0.1, 0.3)	− 17.7 (− 25.1, − 11.0)
High-middle SDI	181.7 (159.6, 202.5)	72.1 (62.9, 82.4)	− 60.3 (− 62.5, − 58.1)	4.9 (4.0, 6.0)	1.3 (1.1, 1.7)	− 72.8 (− 77.8, − 66.0)
Middle SDI	211.2 (179.0, 249.4)	112.8 (95.4, 131.1)	− 46.6 (− 48.7, − 44.1)	21.5 (19.0, 23.3)	4.8 (4.1, 5.4)	− 77.8 (− 80.6, − 74.2)
Low-middle SDI	211.7 (183.4, 248.5)	109.9 (96.0, 123.9)	− 48.1 (− 50.4, − 46.0)	16.3 (13.6, 19.0)	5.8 (4.9, 6.8)	− 64.7 (− 71.1, − 56.1)
Low-SDI	0.0 (0.0, 0.0)	0.0 (0.0, 0.0)	0.0 (0.0, 0.0)	0.0 (0.0, 0.0)	0.0 (0.0, 0.0)	NA
Geographic regions						
Andean Latin America	3328.1 (2887.5, 3875.4)	1224.6 (1089.3, 1362.4)	− 63.2 (− 65.1, − 61.1)	108.7 (80.3, 145.6)	28.9 (21.2, 39.1)	− 73.4 (− 81.8, − 59.1)
Australasia	11.1 (9.7, 12.5)	4.6 (3.9, 5.4)	− 58.2 (− 60.9, − 55.7)	0.11 (0.08, 0.17)	0.04 (0.03, 0.07)	− 61.7 (− 64.8, − 59.1)
Caribbean	6.8 (5.9, 7.8)	3.7 (3.2, 4.3)	− 44.8 (− 48.1, − 41.6)	1.7 (1.2, 2.3)	0.04 (0.03, 0.06)	− 97.4 (− 98.4, − 95.7)
Central Asia	0.0 (0.0, 0.0)	0.0 (0.0, 0.0)	0.0 (0.0, 0.0)	0.0 (0.0, 0.0)	0.0 (0.0, 0.0)	NA
Central Europe	0.0 (0.0, 0.0)	0.0 (0.0, 0.0)	0.0 (0.0, 0.0)	0.0 (0.0, 0.0)	0.0 (0.0, 0.0)	NA
Central Latin America	1516.2 (1277.2, 1810.2)	791.0 (663.3, 920.2)	− 47.8 (− 50.0, − 45.0)	45.0 (35.0, 54.4)	17.6 (13.7, 22.6)	− 60.9 (− 69.1, − 50.9)
Central sub-Saharan Africa	0.0 (0.0, 0.0)	0.0 (0.0, 0.0)	0.0 (0.0, 0.0)	0.0 (0.0, 0.0)	0.0 (0.0, 0.0)	NA
East Asia	0.0 (0.0, 0.0)	0.0 (0.0, 0.0)	0.0 (0.0, 0.0)	0.0 (0.0, 0.0)	0.0 (0.0, 0.0)	NA
Eastern Europe	0.0 (0.0, 0.0)	0.0 (0.0, 0.0)	0.0 (0.0, 0.0)	0.0 (0.0, 0.0)	0.0 (0.0, 0.0)	NA
Eastern sub-Saharan Africa	0.0 (0.0, 0.0)	0.0 (0.0, 0.0)	0.0 (0.0, 0.0)	0.0 (0.0, 0.0)	0.0 (0.0, 0.0)	NA
High-income Asia Pacific	0.8 (0.7, 1.0)	2.5 (2.1, 2.9)	192.9 (174.5, 210.6)	0.01 (0.01, 0.02)	0.02 (0.01, 0.03)	115.9 (93.6, 137.3)
High-income North America	56.9 (47.6, 67.6)	55.9 (46.5, 65.7)	− 1.9 (− 6.2, 3.5)	0.6 (0.4, 0.9)	0.5 (0.3, 0.8)	− 14.2 (− 20.7, − 7.5)
North Africa and Middle East	0.0 (0.0, 0.0)	0.0 (0.0, 0.0)	0.0 (0.0, 0.0)	0.0 (0.0, 0.0)	0.0 (0.00, 0.0)	NA
Oceania	0.0 (0.0, 0.0)	0.0 (0.0, 0.0)	0.0 (0.0, 0.0)	0.0 (0.0, 0.0)	0.0 (0.0, 0.0)	NA
South Asia	0.0 (0.0, 0.0)	0.0 (0.0, 0.0)	0.0 (0.0, 0.0)	0.0 (0.0, 0.0)	0.0 (0.0, 0.0)	NA
Southeast Asia	0.0 (0.0, 0.0)	0.0 (0.0, 0.0)	0.0 (0.0, 0.0)	0.0 (0.0, 0.0)	0.0 (0.0, 0.0)	NA
Southern Latin America	3944.8 (3466.5, 4402.7)	1346.1 (1176.1, 1538.4)	− 65.9 (− 67.7, − 63.9)	86.6 (67.6, 108.4)	23.5 (17.9, 31.0)	− 72.8 (− 78.7, − 64.3)
Southern sub-Saharan Africa	0.0 (0.0, 0.0)	0.0 (0.0, 0.0)	0.0 (0.0, 0.0)	0.0 (0.0, 0.0)	0.0 (0.0, 0.0)	NA
Tropical Latin America	1602.4 (1368.2, 1868.7)	830.8 (715.1, 945.9)	− 48.2 (− 50.7, − 45.2)	288.5 (255.4, 311.9)	59.3 (52.5, 66.6)	− 79.5 (− 81.9, − 76.1)
Western Europe	3.8 (3.3, 4.4)	8.4 (7.3, 9.5)	120.1 (113.7, 127.1)	0.04 (0.03, 0.06)	0.08 (0.05, 0.11)	97.7 (90.1, 105.6)
Western sub-Saharan Africa	0.0 (0.0, 0.0)	0.0 (0.0, 0.0)	0.0 (0.0, 0.0)	0.0 (0.0, 0.0)	0.0 (0.0, 0.0)	NA

*DALYs* disability-adjusted life years, *GBD* global burden of disease, *SDI* sociodemographic index, *UI* uncertainty interval, *NA* not applicable

For onchocerciasis, there were 19.6 million cases (95% UI: 17.8, 21.7) globally, representing a decrease of 16.3% compared with 1990, and the age-standardized prevalence rate was estimated at 246.2 per 100,000 population (95% UI: 222.7, 273.1 per 100,000 population) in 2021, decreasing by − 44.6% (95% UI: − 46.6, − 42.6%) compared with 1990. Notably, the disease burden was still high in specific areas, such as central sub-Saharan Africa, eastern sub-Saharan Africa and western sub-Saharan Africa, reflecting a certain distribution pattern (Table 6). Onchocerciasis was most prevalent across the GBD central sub-Saharan Africa, with 9857.7 per 100,000 population (95% UI: 9222.2, 10,576.1 per 100,000 population) in 2021 and a decrease from 18,426.1 per 100,000 population (95% UI: 17,260.6, 19,762.9 per 100,000 population) in 1990. Over the past 30 years, the age-standardized prevalence rate decreased in the middle-SDI regions (% change = − 68.1, 95% UI: − 74.3, − 61.5), low-middle-SDI regions (% change = − 73.2, 95% UI: − 76.4, − 70.1), and low-SDI regions (% change = − 59.3, 95% UI: − 59.9, − 58.7). The age-standardized prevalence rate of low-SDI regions was 1728.6 per 100,000 population (95% UI: 1593.1, 1892.1 per 100,000 population) in 2021, which was still the highest across all SDI levels. The estimated global age-standardized DALYs rate of onchocerciasis was 15.8 per 100,000 population (95% UI: 9.4, 23.9 per 100,000 population), with a percentage change of − 40.8% (95% UI: − 45.0, − 37.1%) compared with that in 1990, but there was no upwards or downwards trend (*P* > 0.05). From 1990–2021, the estimated global age-standardized DALYs rate of onchocerciasis declined in the middle-SDI regions (% change = − 61.7, 95% UI: − 72.0, − 50.4), low-middle-SDI regions (% change = − 66.3, 95% UI: − 71.9, − 60.6), and low-SDI regions (% change = − 56.9, 95% UI: − 58.2, − 55.4), and there was a pronounced downwards trend in the low-middle-SDI regions, (*P* < 0.05) (Table 6).

**Table 6:** Age-standardized prevalence rates and DALY rates for onchocerciasis in 1990 and 2021 and the percentage change in the age-standardized rates per 100,000 population by GBD region from 1990–2021

Onchocerciasis	Prevalence			DALY		
	Age-standardized prevalence rate per 100,000 population (95% UI) 1990	Age-standardized prevalence rate per 100,000 population (95% UI) 2021	% change in age-standardized prevalence rate 1990–2021	Age-standardized DALY rate per 100,000 population (95% UI) 1990	Age-standardized DALY rate per 100,000 population (95% UI) 2021	% change in age-standardized DALY rate 1990–2021
Global	444.5 (409.4, 485.6)	246.2 (222.7, 273.1)	− 44.6 (− 46.6, − 42.6)	26.7 (16.0, 40.0)	15.8 (9.4, 23.9)	− 40.8 (− 45.0, − 37.1)
SDI groups						
High SDI	0.0 (0.0, 0.0)	0.0 (0.0, 0.0)	0.0 (0.0, 0.0)	0.0 (0.0, 0.0)	0.0 (0.0, 0.0)	NA
High-middle SDI	0.0 (0.0, 0.0)	0.0 (0.0, 0.0)	0.0 (0.0, 0.0)	0.0 (0.0, 0.0)	0.0 (0.0, 0.0)	NA
Middle SDI	80.8 (71.3, 92.8)	25.8 (20.5, 32.0)	− 68.1 (− 74.3, − 61.5)	4.9 (3.0, 7.3)	1.9 (1.0, 3.1)	− 61.7 (− 72.0, − 50.4)
Low-middle SDI	466.1 (431.4, 510.3)	124.7 (107.6, 143.6)	− 73.2 (− 76.4, − 70.1)	28.2 (17.9, 41.1)	9.5 (5.6, 14.4)	− 66.3 (− 71.9, − 60.6)
Low-SDI	4242.8 (3924.3, 4629.1)	1728.6 (1593.1, 1892.1)	− 59.3 (− 59.9, − 58.7)	263.6 (160.5, 394.3)	113.6 (69.4, 169.1)	− 56.9 (− 58.2, − 55.4)
Geographic regions						
Andean Latin America	48.2 (42.3, 53.1)	0.0 (0.0, 0.0)	− 100.0 (− 100.0, − 100.0)	3.5 (2.4, 5.1)	0.0 (0.0, 0.0)	− 100.0 (− 100.0, − 100.0)
Australasia	0.0 (0.0, 0.0)	0.0 (0.0, 0.0)	0.0 (0.0, 0.0)	0.0 (0.0, 0.0)	0.0 (0.0, 0.0)	NA
Caribbean	0.0 (0.0, 0.0)	0.0 (0.0, 0.0)	0.0 (0.0, 0.0)	0.0 (0.0, 0.0)	0.0 (0.0, 0.0)	NA
Central Asia	0.0 (0.0, 0.0)	0.0 (0.0, 0.0)	0.0 (0.0, 0.0)	0.0 (0.0, 0.0)	0.0 (0.0, 0.0)	NA
Central Europe	0.0 (0.0, 0.0)	0.0 (0.0, 0.0)	0.0 (0.0, 0.0)	0.0 (0.0, 0.0)	0.0 (0.0, 0.0)	NA
Central Latin America	193.6 (176.4, 203.8)	1.9 (0.9, 3.4)	− 99.0 (− 99.6, − 98.2)	14.2 (9.9, 19.6)	0.2 (0.1, 0.3)	− 98.8 (− 99.5, − 97.8)
Central sub-Saharan Africa	18,426.1 (17,260.6, 19,762.9)	9857.7 (9222.9, 10,576.1)	− 46.5 (− 47.3, − 45.8)	1129.0 (722.6, 1657.7)	627.2 (400.7, 918.2)	− 44.4 (− 47.8, − 40.9)
East Asia	0.0 (0.0, 0.0)	0.0 (0.0, 0.0)	0.0 (0.0, 0.0)	0.0 (0.0, 0.0)	0.0 (0.0, 0.0)	NA
Eastern Europe	0.0 (0.0, 0.0)	0.0 (0.0, 0.0)	0.0 (0.0, 0.0)	0.0 (0.0, 0.0)	0.0 (0.0, 0.0)	NA
Eastern sub-Saharan Africa	2204.2 (1967.7, 2484.9)	890.3 (801.4, 998.8)	− 59.6 (− 62.0, − 57.0)	133.9 (83.7, 199.5)	63.0 (38.2, 95.3)	− 53.0 (− 57.7, − 47.7)
High-income Asia Pacific	0.0 (0.0, 0.0)	0.0 (0.0, 0.0)	0.0 (0.0, 0.0)	0.0 (0.0, 0.0)	0.0 (0.0, 0.0)	NA
High-income North America	0.0 (0.0, 0.0)	0.0 (0.0, 0.0)	0.0 (0.0, 0.0)	0.0 (0.0, 0.0)	0.0 (0.0, 0.0)	NA
North Africa and Middle East	35.2 (31.5, 39.4)	11.6 (10.5, 12.8)	− 67.1 (− 67.6, − 66.6)	2.3 (1.5, 3.4)	0.7 (0.4, 1.1)	− 68.4 (− 71.9, − 64.7)
Oceania	0.0 (0.0, 0.0)	0.0 (0.0, 0.0)	0.0 (0.0, 0.0)	0.0 (0.0, 0.0)	0.0 (0.0, 0.0)	NA
South Asia	0.0 (0.0, 0.0)	0.0 (0.0, 0.0)	0.0 (0.0, 0.0)	0.0 (0.0, 0.0)	0.0 (0.0, 0.0)	NA
Southeast Asia	0.0 (0.0, 0.0)	0.0 (0.0, 0.0)	0.0 (0.0, 0.0)	0.0 (0.0, 0.0)	0.0 (0.0, 0.0)	NA
Southern Latin America	0.0 (0.0, 0.0)	0.0 (0.0, 0.0)	0.0 (0.0, 0.0)	0.0 (0.0, 0.0)	0.0 (0.0, 0.0)	NA
Southern sub-Saharan Africa	0.0 (0.0, 0.0)	0.0 (0.0, 0.0)	0.0 (0.0, 0.0)	0.0 (0.0, 0.0)	0.0 (0.0, 0.0)	NA
Tropical Latin America	13.7 (11.8, 15.0)	0.1 (0.0, 0.4)	− 99.3 (− 99.9, − 97.3)	1.0 (0.7, 1.4)	0.01 (0.00, 0.03)	− 99.2 (− 99.9, − 96.8)
Western Europe	0.0 (0.0, 0.0)	0.0 (0.0, 0.0)	0.0 (0.0, 0.0)	0.0 (0.0, 0.0)	0.0 (0.0, 0.0)	NA
Western sub-Saharan Africa	7585.9 (6921.4, 8396.0)	1393.6 (1193.6, 1636.7)	− 81.6 (− 83.5, − 79.9)	475.1 (286.1, 714.2)	106.5 (60.8, 164.0)	− 77.6 (− 79.8, − 75.3)

*DALYs* disability adjusted life years, *GBD* global burden of disease, *SDI* sociodemographic index, *UI* uncertainty interval, *NA* not applicable

### Association between the burden of the vb-pIDP and the SDI

**Fig. 1:**
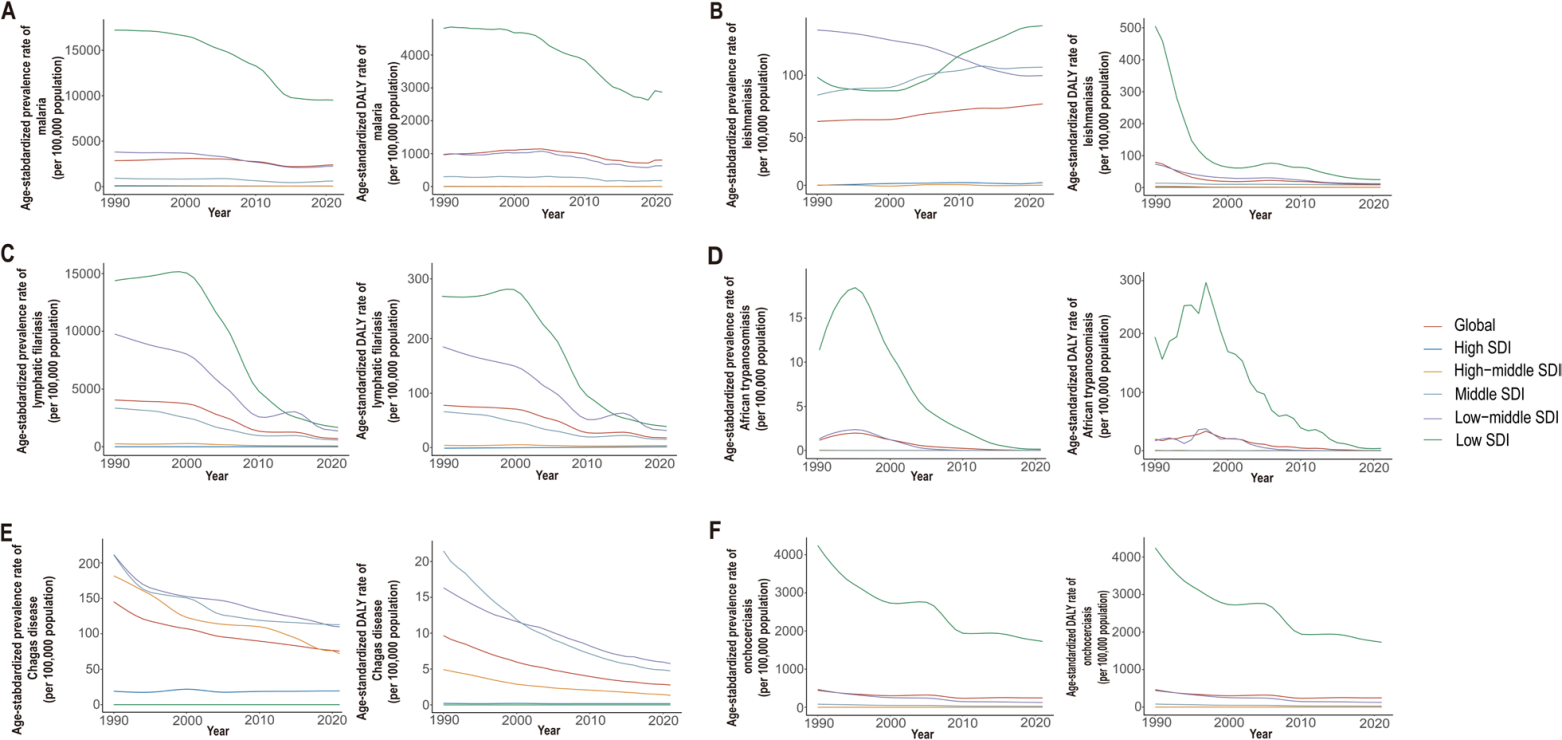
Age-standardized prevalence rate and DALYs rate of vb-pIDP globally and across all SDI quintiles, 1990–2021. **A** Malaria. **B** Leishmaniasis. **C** Lymphatic filariasis. **D** African trypanosomiasis. **E** Chagas disease. **F** Onchocerciasis. *SDI* sociodemographic index, *Vb-IDP* vector-borne infectious disease of poverty

Overall, over the past 30 years, the age-standardized prevalence rate and DALYs rate for malaria, leishmaniasis, lymphatic filariasis, African trypanosomiasis, Chagas disease, and onchocerciasis have shown declining trends globally and in regions with different SDI levels (Fig. 1).

**Fig. 2:**
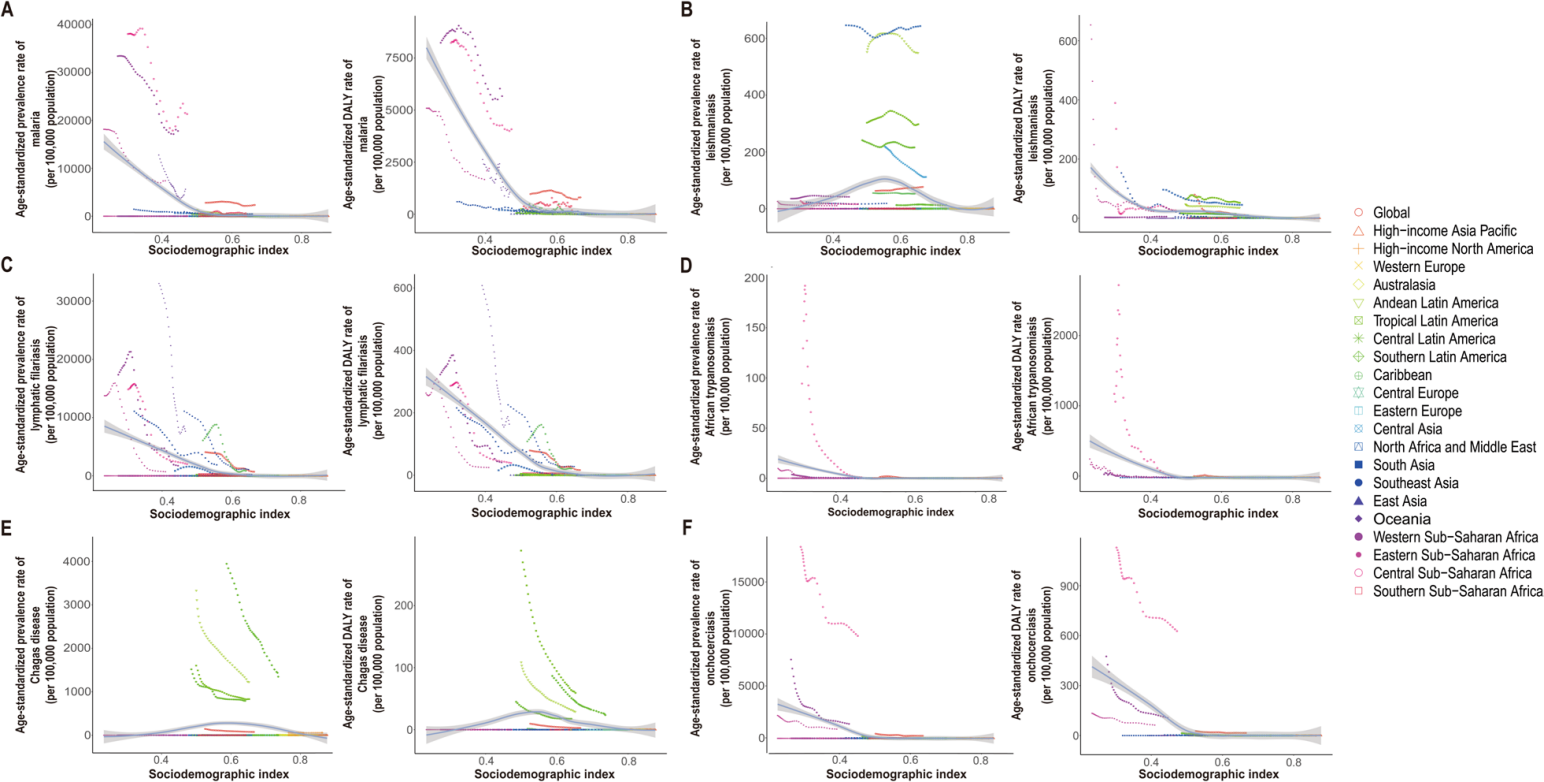
Age-standardized prevalence rate and DALYs rate of vb-pIDP, globally and for 21 GBD regions, by SDI, 1990–2021. **A** Malaria. **B** Leishmaniasis. **C** Lymphatic filariasis. **D** African trypanosomiasis. **E** Chagas disease. **F** Onchocerciasis. Every dot represents a specific year between 1900 and 2021, and its color implies the geographic region. *SDI* sociodemographic index, *Vb-IDP* vector-borne infectious disease of poverty

For malaria, the age-standardized prevalence rate and DALYs rate have decreased globally and across all SDI levels, with the most significant decline occurring in low-SDI regions. In the low-SDI regions, the age-standardized prevalence decreased from 17,155.4 per 100,000 population (95% UI: 15,844.1, 18,797.5 per 100,000 population) in 1990 to 9458.6 per 100,000 population (95% UI: 8494.2, 10,473.8 per 100,000 population) in 2021, whereas the age-standardized DALYs rate decreased from 4811.5 per 100,000 population (95% UI: 2580.7, 8569.1 per 100,000 population) in 1990 to 2869.2 per 100,000 population (95% UI: 1107.4, 5683.9 per 100,000 population) in 2021 and was still significantly higher than that in other SDI level regions (Fig. 1A, Table 1). Over the past 30 years, the age-standardized prevalence rates were higher than expected on the basis of the SDI at the global level and in central sub-Saharan Africa, Oceania, western sub-Saharan Africa, and eastern sub-Saharan Africa. Additionally, the age-standardized DALYs rates were higher than expected on the basis of the SDI at the global level and in central sub-Saharan Africa, western sub-Saharan Africa, and southern sub-Saharan Africa during the same period (Fig. 2A).

For leishmaniasis, the overall age-standardized prevalence rate fluctuated from 1990–2021. The age-standardized prevalence rate decreased only in the high-SDI regions, whereas it increased globally and, in the middle-SDI and low-SDI regions (Fig. 1B). Overall, the age-standardized DALYs rate for leishmaniasis decreased globally from 1990–2021. In particular, there was a higher age-standardized DALYs rate in the low-SDI regions than in the other regions in 1990, but it significantly declined over time and stabilized by 2021. The age-standardized DALYs rate of low-SDI regions decreased by − 95.0% (95% UI: − 96.8, − 91.3) (Table 2). In other regions, the age-standardized DALYs rates were relatively low and gradually decreased. Over the past 30 years, the age-standardized prevalence rates were higher than expected on the basis of the SDI in North Africa and the Middle East, Andean Latin America, tropical Latin America, central Latin America, and central Asia. Additionally, the age-standardized DALYs rates were higher than expected on the basis of the SDI in tropical Latin America, North Africa, the Middle East, and Andean Latin America (Fig. 2B).

From 1990–2021, the age-standardized prevalence rate and DALYs rate for lymphatic filariasis declined globally, as did the rates in the low-SDI, low-middle-SDI, and middle-SDI regions, with some fluctuations (Fig. 1C). The age-standardized prevalence rate and DALYs rate of lymphatic filariasis decreased most significantly in the low-SDI regions, followed by the low-middle-SDI regions. The age-standardized prevalence rate decreased by − 88.4% (95% UI: − 90.9, − 85.7) and the age-standardized DALYs rate decreased by − 86.6% (95% UI: − 90.1, − 80.8) in low-SDI regions (Table 3). However, the age-standardized prevalence rate and DALYs rate remained extremely low and stable in high-SDI and high-middle-SDI regions. Additionally, the age-standardized prevalence rate and DALYs rate were higher than expected on the basis of the SDI at the global level and in Oceania, southeast Asia, the Caribbean, South Asia, and western sub-Saharan Africa (Fig. 2C).

For African trypanosomiasis, the age-standardized prevalence rate and DALYs rate rapidly increased after 1990, peaked in approximately 1996, then significantly declined and stabilized by 2021 in low-SDI regions. In contrast, the age-standardized prevalence rate and DALYs rate were relatively low in other regions, with overall gradual but declining trends (Fig. 1D). From 1990–2021, the age-standardized prevalence rate and DALYs rate in central sub-Saharan Africa were significantly higher than expected (Fig. 2D). The age-standardized prevalence rate of African trypanosomiasis was 94.4 per 100,000 population (95% UI: 41.7, 183.2) compared with the global average of 1.2 per 100,000 population (95% UI: 0.5, 2.2) in 1990, and the age-standardized prevalence rate was 0.9 per 100,000 population (95% UI: 0.5, 1.6) compared with the global average of 0.03 per 100,000 population (95% UI: 0.01, 0.06) in 2021. The age-standardized DALYs rate of African trypanosomiasis was 1181.1 per 100,000 population (95% UI: 585.3, 1964.8) compared with the global average of 19.9 per 100,000 population (95% UI: 10.0, 31.9), and the age-standardized DALYs rate was 15.0 per 100,000 population (95% UI: 7.4, 25.9) compared with the global average of 0.8 per 100,000 population (95% UI: 0.3, 2.3) (Table 4).

Over the past 30 years, both the age-standardized prevalence rate and DALYs rate of Chagas disease have declined globally, as have those of high–middle-SDI, middle-SDI, and low–middle-SDI regions, and stabilized by 2021 (Fig. 1E). From 1990–2021, the age-standardized prevalence rate and DALYs rate in southern Latin America, tropical Latin America, Andean Latin America, and central Latin America were significantly higher than expected (Fig. 2E). For example, the age-standardized prevalence of Central Latin America was 1516.2 per 100,000 population (95% UI: 1277.2, 1810.2) compared with the global average of 145.2 per 100,000 population (95% UI: 125.4, 168.3) in 1990, and the age-standardized prevalence rate was 791.0 per 100,000 population (95% UI: 663.3, 920.2) compared with 75.6 per 100,000 population (95% UI: 65.2, 86.5) in 2021. The age-standardized DALYs rate of Central Latin America was 14.2 per 100,000 population (95% UI: 9.9, 19.6) compared with the global average of 26.7 per 100,000 population (95% UI: 16.0, 40.0) in 1990, and the age-standardized DALYs rate was 0.2 per 100,000 population (95% UI: 0.1, 0.3) compared with the global average of 15.8 per 100,000 population (95% UI: 9.4, 23.9) in 2021 (Table 5). The global age-standardized prevalence and DALYs rate for onchocerciasis have declined and fluctuated, with the most significant decrease observed. The age-standardized prevalence rate of onchocerciasis decreased by − 0.6% (95% UI: − 0.6, − 0.6) and the age-standardized DALYs rate decreased by − 0.57% (95% UI: − 0.58, − 0.55) (Fig. 1F, Table 6). Additionally, the age-standardized prevalence rates were higher than expected on the basis of the SDI in southeast Asia and central sub-Saharan Africa, and the age-standardized DALYs rate in central sub-Saharan Africa was higher than expected (Fig. 2F).

**Fig. 3:**
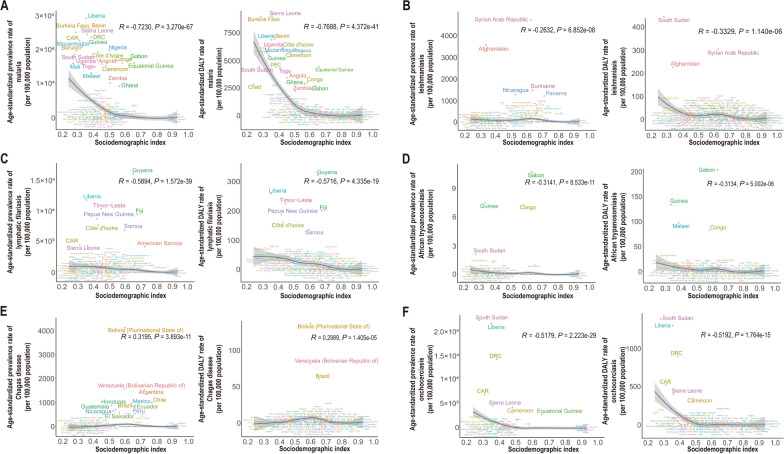
Age-standardized prevalence rate and DALYs rate of vb-pIDP in 204 countries with different SDI levels in 2021. **A** Malaria. **B** Leishmaniasis. **C** Lymphatic filariasis. **D** African trypanosomiasis. **E** Chagas disease. **F** Onchocerciasis. Every dot represents the prevalence rate of a country, and its color has been chosen arbitrarily without any implications for the geographic region. *CAR* Central African Republic, *DRC* Democratic Republic of the Congo, *SDI* sociodemographic index, *Vb-pIDP* vector-borne infectious disease of poverty

In 2021, there were negative correlations between the age-standardized prevalence rates of malaria (*R* = − 0.7230, *P* = 3.270e−67), leishmaniasis (*R* = − 0.2632, *P* = 6.852e−08), African trypanosomiasis (*R* = − 0.3141, *P* = 8.533e−11), lymphatic filariasis (*R* = − 0.5894, *P* = 1.572e−39), and onchocerciasis (*R* = − 0.5179, *R* = 2.223e−29) and the SDI, with the prevalence decreasing significantly as the SDI increased (Fig. 3A–D, F). There were negative correlations between the age-standardized DALYs rates for malaria (*R* = − 0.7688, *P* = 4.372e−41), leishmaniasis (*R* = − 0.3329, *P* = 1.140e−06), African trypanosomiasis (*R* = − 0.3134, *P* = 5.002e−06), lymphatic filariasis (*R* = − 0.5716, *P* = 4.335e−19), and onchocerciasis (*R* = − 0.5192, *P* = 1.764e−15) and the SDI, with the burden decreasing significantly as the SDI increased (Fig. 3A–D, F). However, for Chagas disease, there were positive correlations between the age-standardized prevalence (*R* = 0.3195, *P* = 3.893e−11) and DALYs (*R* = 0.2989, *P* = 14.05e−05) rates and the SDI (Fig. 3E).

### Gender and age distribution for the vb-pIDP

**Fig. 4:**
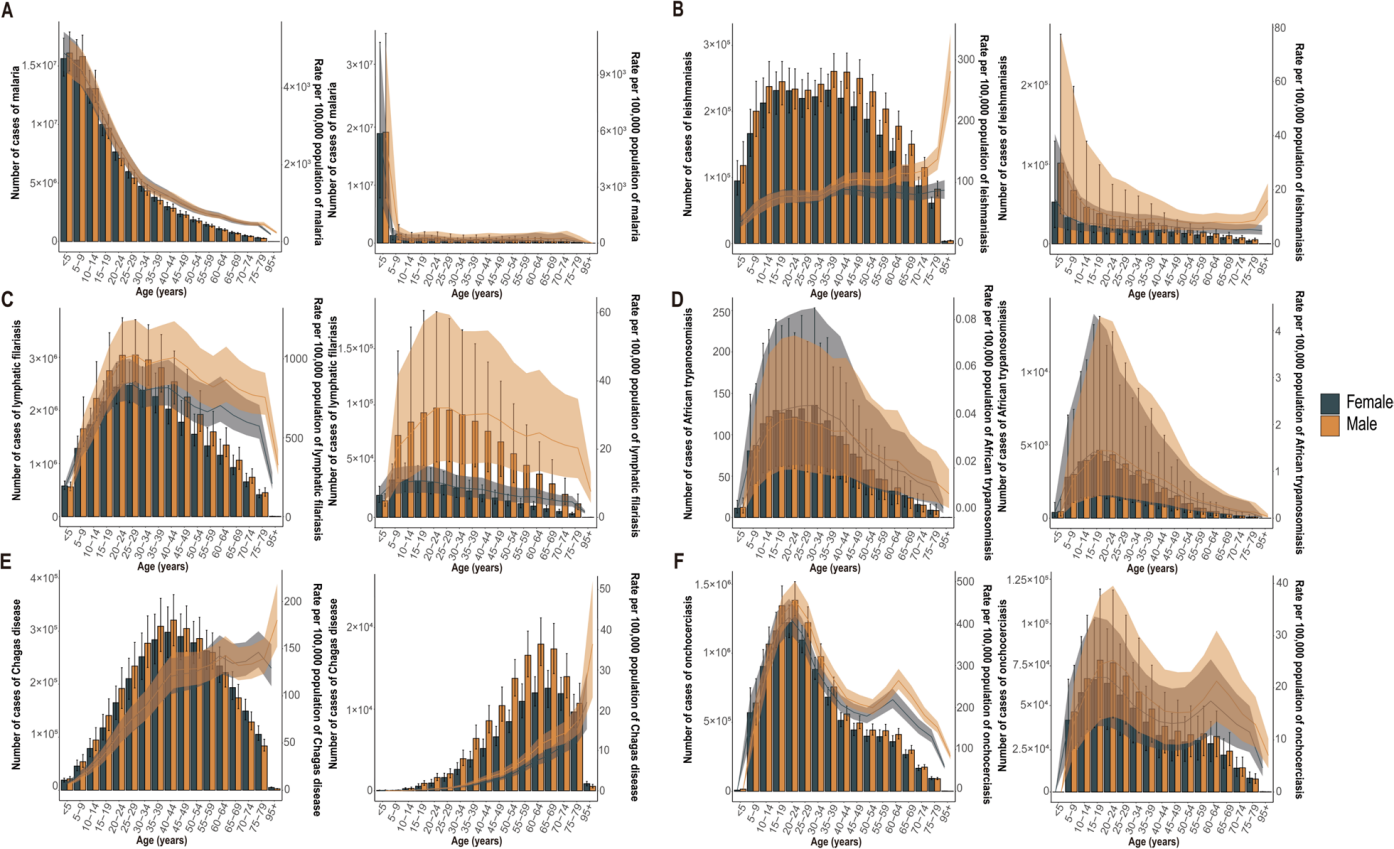
Age-standardized prevalence and DALYs rate of vb-pIDP in different age groups in 2021. **A** Malaria. **B** Leishmaniasis. **C** Lymphatic filariasis. **D** African trypanosomiasis. **E** Chagas disease. **F** Onchocerciasis. *SDI* sociodemographic index, *Vb-pIDP* vector-borne infectious diseases of poverty, *DALYs* disability-adjusted life years.

The specific prevalence rates for malaria, leishmaniasis, African trypanosomiasis, Chagas disease, and onchocerciasis and the specific DALYs rates for malaria, leishmaniasis, African trypanosomiasis, and onchocerciasis were not different between males and females across all age groups (*P* > 0.05)** (**Fig. 4A, B, D–F). For lymphatic filariasis, in the 15–19 years age group and above, the specific DALYs rates were higher in females than in males (*P* < 0.05), with no differences in the other age groups (*P* > 0.05) (Fig. 4C). Conversely, in the 35–39, 40–44, 45–49, 50–54, 55–59, and 60–64 age groups, the specific DALYs rates for Chagas disease were higher in males than in females (*P* < 0.05), with no differences in the other age groups (*P* > 0.05) (Fig. 4C).

The specific prevalence rate and DALYs rate of malaria were highest in children under five years of age and decreased with increasing age (Fig. 4A). The number of cases and DALYs rate of lymphatic filariasis peaked in the 20–24 years age group and then gradually declined with increasing age, and the number of malaria cases and DALYs rate peaked in the 15–19 years age group and then gradually declined with increasing age (Fig. 4C). For African trypanosomiasis, overall, the DALYs rate was relatively low in children under the age of five years, started to gradually increase from the age of 5 years, peaked at 20–24 years, then gradually decreased, and then continued to decline with increasing age (Fig. 4D).

### Variance analysis in 21 geographic regions of the globe

In 2021, among the six vb-pIDP, malaria had the highest age-standardized prevalence rate and DALYs rate globally, followed by lymphatic filariasis. Furthermore, malaria had the highest age-standardized prevalence and DALYs rate in western sub-Saharan Africa and central sub-Saharan Africa, followed by eastern sub-Saharan Africa and Oceania (Additional file 1: Figure S1). Notably, lymphatic filariasis had the highest age-standardized prevalence rate in Oceania, whereas onchocerciasis had the highest age-standardized prevalence rate in central sub-Saharan Africa (Additional file 1: Figure S1A). The age-standardized DALYs rate of onchocerciasis was higher in central sub-Saharan Africa than in other regions of the world (Additional file 1: Figure S1B).

### Risk factors for malaria

**Fig. 5:**
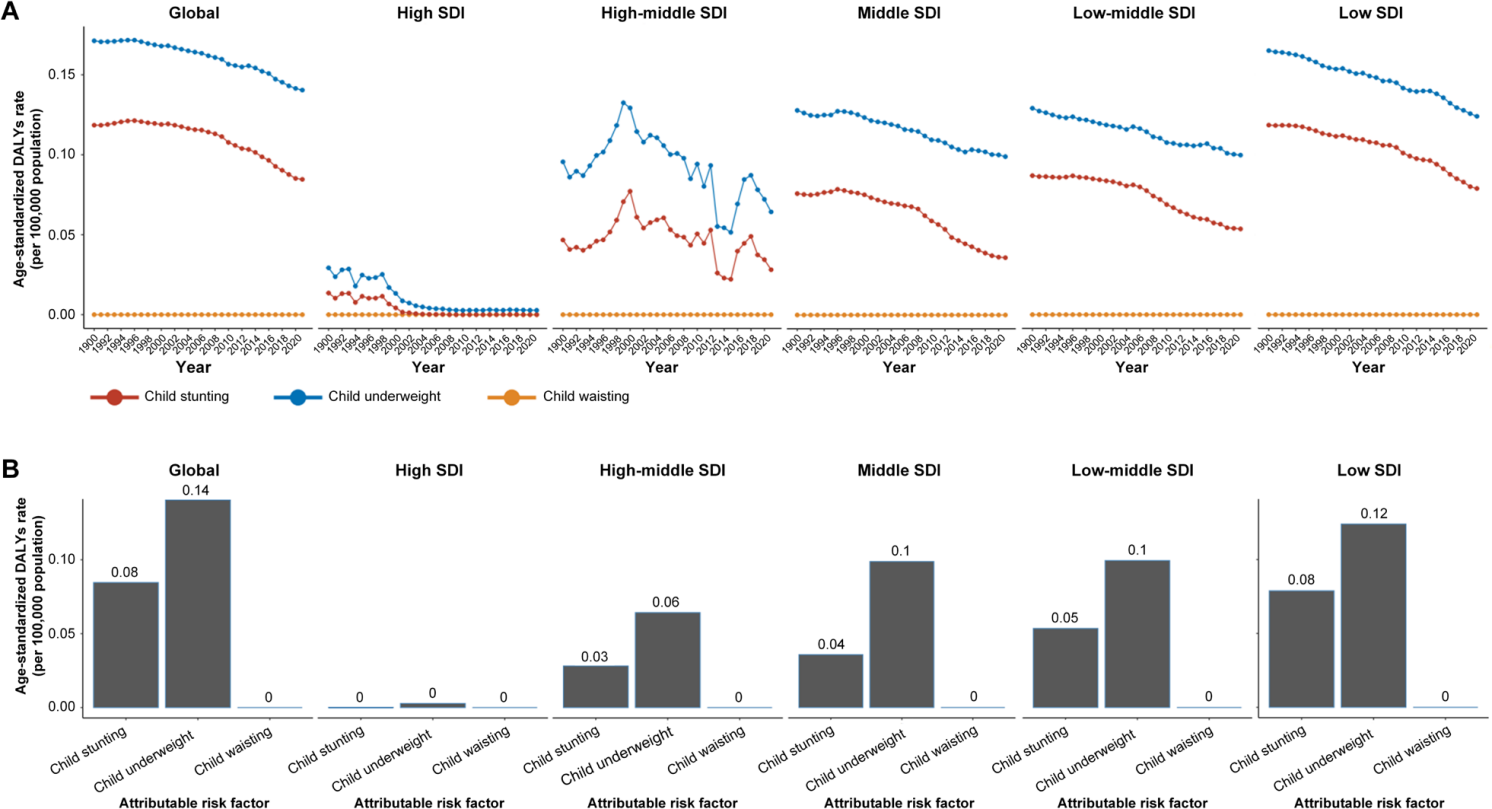
The burden of malaria attributable to risk factors (child stunting, child underweight, and child wasting) across SDI quintiles. **A** In 1990**–**2021. **B** In 2021. SDI: sociodemographic index

The GBD 2021 assessed the contributions of three risk factors for malaria-related DALYs. Among these risk factors, the leading risk factor was child underweight, and the second risk factor was child stunting. Globally, the impact of child underweight and child stunting showed a declining trend, whereas the impact of child wasting remained negligible, and 0.14% of DALYs related to malaria were attributed to child underweight, whereas 0.08% of DALYs related to malaria were attributed to child stunting (Fig. 5). In high-SDI regions, the impacts of child underweight and child stunting on malaria DALYs are very small and decrease annually, with child wasting having almost no impact. In high-middle-SDI regions, the impact fluctuated but showed an overall decline, with child underweight having a slightly greater impact than child stunting (Fig. 5A). In the middle-SDI regions, the impacts also showed a declining trend. In the low-middle- and low-SDI regions, the impacts were the greatest, yet they also exhibited a downwards trend (Fig. 5B). Overall, the impacts of child underweight and child stunting on malaria DALYs were greatest in low-SDI regions and minimal in high-SDI regions, with these impacts showing an overall decline from 1990–2021.

## Discussion

### Global burden of disease and sociodemographic index

This study provides comprehensive estimates of the prevalence and DALYs of malaria, leishmaniasis, lymphatic filariasis, African trypanosomiasis, Chagas disease, and onchocerciasis in different SDI regions, sexes, and age groups globally and regionally over the past 30 years. This study revealed that from 1990–2021, the age-standardized prevalence rate and DALYs rate for notable vb-pIDP were very low in high-SDI regions. In contrast, in the middle-SDI, low-middle-SDI, and low-SDI regions, some vb-pIDP, especially malaria, presented higher age-standardized prevalence rate and DALYs rate. Additionally, there were negative correlations between the age-standardized prevalence rate and DALYs rate for malaria, leishmaniasis, lymphatic filariasis, African trypanosomiasis, onchocerciasis and SDI. This finding indicated that improving socioeconomic and development levels plays a crucial role in reducing the burden, especially for low-SDI countries, such as Gabon, Guinea, Congo, Malawi, South Sudan, Syria, Afghanistan, Guyana, Liberia, Timor-Leste, Papua New Guinea, Côte d’Ivoire, Burkina Faso, Sierra Leone, Benin, and Congo. There are several potential explanations for this phenomenon. First, low-SDI areas typically lack good sanitation facilities and infrastructure, such as clean water sources, sanitation facilities, and health education, which increases the risk of parasite transmission and infection [15]. Second, medical resources, including medical facilities, healthcare personnel, and medication supplies, may be limited in low-SDI areas, leading to insufficient prevention, treatment, and control of the vb-pIDP [21]. Additionally, environmental factors in low-SDI areas may be more conducive to parasite transmission [22, 23], such as inappropriate climate conditions and water resource management, which can further increase the risk of vb-pIDP, thus exacerbating the burden in these areas. The continual emergence of novel zoonotic diseases poses great challenges to human disease prevention and control, necessitating the continuous improvement of disease monitoring systems to proactively prevent and manage outbreaks [24, 25].

### Age and gender-specific vulnerabilities are associated with vb-pIDP

On the one hand, children are likely susceptible to increased disease prevalence and burden. Children have relatively weaker immune systems and are more susceptible to several vb-pIDP, such as malaria and leishmaniasis, leading to a greater disease burden in this age group population [26]. Moreover, since children often engage in outdoor activities, they have more opportunities to come into contact with vectors (such as mosquitoes and ticks), thereby increasing the risk of contracting vb-pIDP [27]. When children contract vb-pIDP, it can lead to higher hospitalization and mortality rates, thereby increasing the economic burden on families and society [28]. On the other hand, older individuals with compromised immune function, such as those with Chagas disease, are more prone to vb-pIDP [29–31]. Notably, the DALYs of Chagas disease and lymphatic filariasis were greater in males than in females across different age groups. Men typically engage in outdoor activities or work in rural areas more frequently than women do, which increases their likelihood of encountering vector insects that transmit Chagas disease, such as triatomine bugs. This higher exposure rate may lead to a higher infection rate among men, thereby affecting their DALYs [32]. Therefore, it is crucial to develop targeted public health strategies based on the characteristics of different age groups and genders to effectively alleviate the health burden imposed by these diseases on diverse populations. Preventive and control measures should be taken during the preparation and storage of food and beverages to address Chagas disease, and the hygienic conditions in food processing and storage facilities should be improved [33]. Furthermore, integrating modern technologies and solutions into emerging zoonotic disease monitoring and response systems holds great potential for improving disease control and prevention [34, 35].

### Maternal-child health strategies for malaria prevention

Malnutrition and a lack of health resources significantly exacerbate the disease burden of malaria in different regions and across various levels of social development, severely impacting the health of children and maternal populations. Child underweight and child stunting were significant risk factors for the age-standardized DALYs rate of malaria, with varying impacts across regions and SDI levels. Despite the annual reduction in various risk factors for the age-standardized DALYs rate of malaria, it remains a significant cause of adverse health outcomes for mothers and their offspring [36]. In low-SDI regions, limited resources cause child stunting and child underweight to have a greater impact on the malaria burden because vulnerable children have a weaker immune system. Conversely, high-SDI regions with better access to healthcare and nutrition experience lower impacts of these risk factors on the malaria burden [37]. Prenatal exposure to vector-borne and other infectious diseases may impact children’s early physical, social, emotional, and cognitive development, making pregnant and postpartum women a group that requires more attention [38, 39]. Improved healthcare interventions targeting maternal and child health, including antenatal care and nutrition programs, can significantly reduce malaria prevalence among children in impoverished areas and lower the malaria disease burden [35, 40, 41]. Research indicates that a balanced diet, vaccination, and breastfeeding to enhance the immunity of children can effectively reduce the incidence of malaria in children [42]. Disease control strategies, monitoring and response systems, and diagnostic technologies for malaria are considered the top three priorities for capacity building in African countries [43, 44]. Additionally, the intensification of malaria control initiatives may have made a significant contribution to reducing the burden of malaria in poverty-stricken areas [45].

### One Health strategy for vb-pIDP control

One Health approach offers a holistic framework for addressing the complex challenges of the vb-pIDP [46]. It advocates for interdisciplinary collaboration, encompassing public health, veterinary medicine, ecology, environmental science, and other fields, to address and solve health issues more comprehensively and systematically [47]. For example, immunomics plays a crucial role in the prevention and control of vb-pIDP [48]. Moreover, establishing a comprehensive monitoring system can track the spread of infectious diseases among animals, the environment, and human populations in real time, allowing for early detection and response to potential outbreak risks [34, 49]. Improving the habitat of vector organisms (such as controlling standing water and eliminating mosquito breeding sites), while simultaneously strengthening the construction and maintenance of public health facilities (to increase water quality and sanitation), can reduce opportunities for contact with vectors and help decrease the transmission of vb-pIDP [49]. Vaccinating key animal hosts, such as livestock and wildlife, to reduce their role as reservoirs for pathogens, along with implementing animal disease monitoring and control programs, e.g., deworming and pathogen testing, can effectively reduce the risk of disease transmission to humans [50]. Raising awareness of vb-pIDP, changing poor hygiene practices through education, encouraging the use of preventive measures such as mosquito nets and insecticides, and enhancing medical resource support in impoverished areas by providing early diagnosis and treatment services can effectively reduce disease incidence and mortality rates [51]. Additionally, promoting the development and implementation of comprehensive health policies by the government; supporting the One Health approach, such as funding interdisciplinary research and intervention programs; and mobilizing community residents to participate in health monitoring and preventive measures, e.g., organizing water drainage efforts and health awareness campaigns, can strengthen the capacity to control local vb-pIDP [52, 53].

### Limitations

This study utilized 2021 GBD data to compressively determine recent estimates of the prevalence and subsequent impacts of notable vb-pIDP. These patterns cannot be discovered from the raw data in the database, so further analyses of these data could help relevant departments and individuals better understand and uncover hidden information, find value, support decision-making, optimize resource utilization, and improve control and prevention capabilities. However, this study has several limitations. First, the estimation of the burden of the vb-pIDP depends on the availability and quality of the primary data in the GBD 2021. The raw data from the GBD study 2021 are not available in some countries, especially low- and middle-income countries. Second, the diagnosis and detection of vb-pIDP might have been inconsistent across countries and over time, which might affect the comparability of the results. Third, although this study describes the burdens of six notable vb-pIDP (malaria, leishmaniasis, lymphatic filariasis, African trypanosomiasis, Chagas disease, and onchocerciasis), it does not predict future vb-pIDP data trends.

## Conclusions

Despite a significant decline in the age-standardized prevalence rate and DALYs rate over the past 30 years for the vb-pIDP involved in the present study, malaria remains the highest burden by 2021. Child underweight and child stunting are key risk factors for the age-standardized DALYs rate of malaria, and interventions targeting maternal and child health will be effective in reducing their impact. Moreover, our study revealed a positive correlation between the vb-pIDP and SDI, which was attributed to poor sanitation conditions and limited medical resources. This research provides a comprehensive estimation of the burden of vb-pIDP, offering more epidemiological evidence for future exploration in the prevention and control of such diseases.

## Supplementary Information

Additional file 1

## Data Availability

The data sources and codes used in the GBD, Injuries, and Risk Factors Study 2021 are publicly available at http://ghdx.healthdata.org/gbd-results-tool.

## Abbreviations

ASR: Age-standardized rate
DALYs: Disability adjusted life years
GBD: Global burden of disease
GHDx: Global Health Data Exchange
LOWESS: Local weighted scatterplot smoothing
RECORD: Reporting of studies conducted using observational routinely collected health data
SDI: Sociodemographic index
UI: Uncertainty interval
Vb-pIDP: Vector-borne parasitic infectious diseases of poverty
YLDs: Years lived with disability
YLLs: Years of life lost
