# Supplementary material for: Prevalence and attributable health burdens of vector-borne parasitic infectious diseases of poverty, 1990–2021: findings from the Global Burden of Disease Study 2021
Source: Infect Dis Poverty. 2024 Dec 11;13:96. doi: 10.1186/s40249-024-01260-x (PMC11633012; doi:10.1186/s40249-024-01260-x)
Supplement: Supplementary file 1 — Additional file 1 [file 40249_2024_1260_MOESM1_ESM.docx]

**Prevalence and attributable health burdens of vector-borne parasitic infectious diseases of poverty, 1990 – 2021: findings from the Global Burden of Disease Study 2021**

Yin-Shan Zhu^1^, Zhi-Shan Sun^1^, Jin-Xin Zheng^1^, Shun-Xian Zhang^2^, Jing-Xian Yin^1^, Han-Qing Zhao^1^, Hai-Mo Shen^3^, Gad Baneth^4^, Jun-Hu Chen^3,5*^, Kokouvi Kassegne^1*^

**Additional file 1**

**Table S1. List of International Classification of Diseases (ICD) codes for malaria, leishmaniasis, lymphatic filariasis, African trypanosomiasis, Chagas disease, and onchocerciasis.**

| **Cause Name** | **ICD 10** | **ICD 9** |
| --- | --- | --- |
| malaria | B50-B50.0, B50.8-B52.0, B52.8-B53.1, B53.8-B54.0, P37.3-P37.4 | 084-084.9, V12.03, V75.1 |
| leishmaniasis | B55-B55.9 | 085-085.9, V05.2, V75.2 |
| lymphatic filariasis | B74-B74.2 | 125.0-125.2 |
| African trypanosomiasis | B56-B56.9 | 086.3-086.9, V75.3 |
| Chagas disease | B57-B57.5, K93.1 | 086-086.2, 425.5 |
| onchocerciasis | B73-B73.1 | 125.3 |

**Table S2. Geographic regions, countries or territories in GBD 2021.**

| **Geographic regions** | **Countries or Territories** |
| --- | --- |
| **Andean Latin America** |  |
|  | Bolivia (Plurinational State of) |
|  | Ecuador |
|  | Peruz |
| **Australasia** |  |
|  | Australia |
|  | New Zealand |
| **Caribbean** |  |
|  | Antigua and Barbuda |
|  | Bahamas |
|  | Barbados |
|  | Belize |
|  | Cuba |
|  | Dominica |
|  | Dominican Republic |
|  | Grenada |
|  | Guyana |
|  | Haiti |
|  | Jamaica |
|  | Saint Lucia |
|  | Saint Vincent and the Grenadines |
|  | Suriname |
|  | Trinidad and Tobago |
| **Central Asia** |  |
|  | Armenia |
|  | Azerbaijan |
|  | Georgia |
|  | Kazakhstan |
|  | Kyrgyzstan |
|  | Mongolia |
|  | Tajikistan |
|  | Turkmenistan |
|  | Uzbekistan |
| **Central Europe** |  |
|  | Albania |
|  | Bosnia and Herzegovina |
|  | Bulgaria |
|  | Croatia |
|  | Czechia |
|  | Hungary |
|  | North Macedonia |
|  | Montenegro |
|  | Poland |
|  | Romania |
|  | Serbia |
|  | Slovakia |
|  | Slovenia |
| **Central Latin America** |  |
|  | Colombia |
|  | Costa Rica |
|  | El Salvador |
|  | Guatemala |
|  | Honduras |
|  | Mexico |
|  | Nicaragua |
|  | Panama |
|  | Venezuela (Bolivarian Republic of) |
| **Central Sub-Saharan Africa** |  |
|  | Angola |
|  | Central African Republic |
|  | Congo |
|  | Democratic Republic of the Congo |
|  | Equatorial Guinea |
|  | Gabon |
| **East Asia** |  |
|  | China |
|  | Democratic People's Republic of Korea |
|  | Taiwan (Province of China) |
| **Eastern Europe** |  |
|  | Belarus |
|  | Estonia |
|  | Latvia |
|  | Lithuania |
|  | Republic of Moldova |
|  | Russian Federation |
|  | Ukraine |
| **Eastern Sub-Saharan Africa** |  |
|  | Burundi |
|  | Comoros |
|  | Djibouti |
|  | Eritrea |
|  | Ethiopia |
|  | Kenya |
|  | Madagascar |
|  | Malawi |
|  | Mauritius |
|  | Mozambique |
|  | Rwanda |
|  | Seychelles |
|  | Somalia |
|  | United Republic of Tanzania |
|  | Uganda |
|  | Zambia |
| **High-income Asia Pacific** |  |
|  | Brunei Darussalam |
|  | Japan |
|  | Republic of Korea |
|  | Singapore |
| **High-income North America** |  |
|  | Canada |
|  | United States of America |
| **North Africa and Middle East** |  |
|  | Algeria |
|  | Bahrain |
|  | Egypt |
|  | Iran (Islamic Republic of) |
|  | Iraq |
|  | Jordan |
|  | Kuwait |
|  | Lebanon |
|  | Libya |
|  | Morocco |
|  | Palestine |
|  | Oman |
|  | Qatar |
|  | Saudi Arabia |
|  | Syrian Arab Republic |
|  | Tunisia |
|  | Turkey |
|  | United Arab Emirates |
|  | Yemen |
| **Oceania** |  |
|  | Fiji |
|  | Kiribati |
|  | Marshall Islands |
|  | Micronesia (Federated States of) |
|  | Papua New Guinea |
|  | Samoa |
|  | Solomon Islands |
|  | Tonga |
|  | Vanuatu |
| **South Asia** |  |
|  | Afghanistan |
|  | Bangladesh |
|  | Bhutan |
|  | India |
|  | Nepal |
|  | Pakistan |
| **Southeast Asia** |  |
|  | Cambodia |
|  | Indonesia |
|  | Lao People's Democratic Republic |
|  | Malaysia |
|  | Maldives |
|  | Myanmar |
|  | Philippines |
|  | Sri Lanka |
|  | Thailand |
|  | Timor-Leste |
|  | Viet Nam |
| **Southern Latin America** |  |
|  | Argentina |
|  | Chile |
|  | Uruguay |
| **Southern Sub-Saharan Africa** |  |
|  | Botswana |
|  | Lesotho |
|  | Namibia |
|  | South Africa |
|  | Eswatini |
|  | Zimbabwe |
| **Tropical Latin America** |  |
|  | Brazil |
|  | Paraguay |
| **Western Europe** |  |
|  | Andorra |
|  | Austria |
|  | Belgium |
|  | Cyprus |
|  | Denmark |
|  | Finland |
|  | France |
|  | Germany |
|  | Greece |
|  | Iceland |
|  | Ireland |
|  | Israel |
|  | Italy |
|  | Luxembourg |
|  | Malta |
|  | Netherlands |
|  | Norway |
|  | Portugal |
|  | Spain |
|  | Sweden |
|  | Switzerland |
|  | United Kingdom |
| **Western Sub-Saharan Africa** |  |
|  | Benin |
|  | Burkina Faso |
|  | Cameroon |
|  | Cabo Verde |
|  | Chad |
|  | Côte d'Ivoire |
|  | Gambia |
|  | Ghana |
|  | Guinea |
|  | Guinea-Bissau |
|  | Liberia |
|  | Mali |
|  | Mauritania |
|  | Niger |
|  | Nigeria |
|  | Sao Tome and Principe |
|  | Senegal |
|  | Sierra Leone |
|  | Togo |
|  | American Samoa |
|  | Bermuda |
|  | Cook Islands |
|  | Greenland |
|  | Guam |
|  | Monaco |
|  | Nauru |
|  | Niue |
|  | Northern Mariana Islands |
|  | Palau |
|  | Puerto Rico |
|  | Saint Kitts and Nevis |
|  | San Marino |
|  | Tokelau |
|  | Tuvalu |
|  | United States Virgin Islands |
|  | South Sudan |
|  | Sudan |


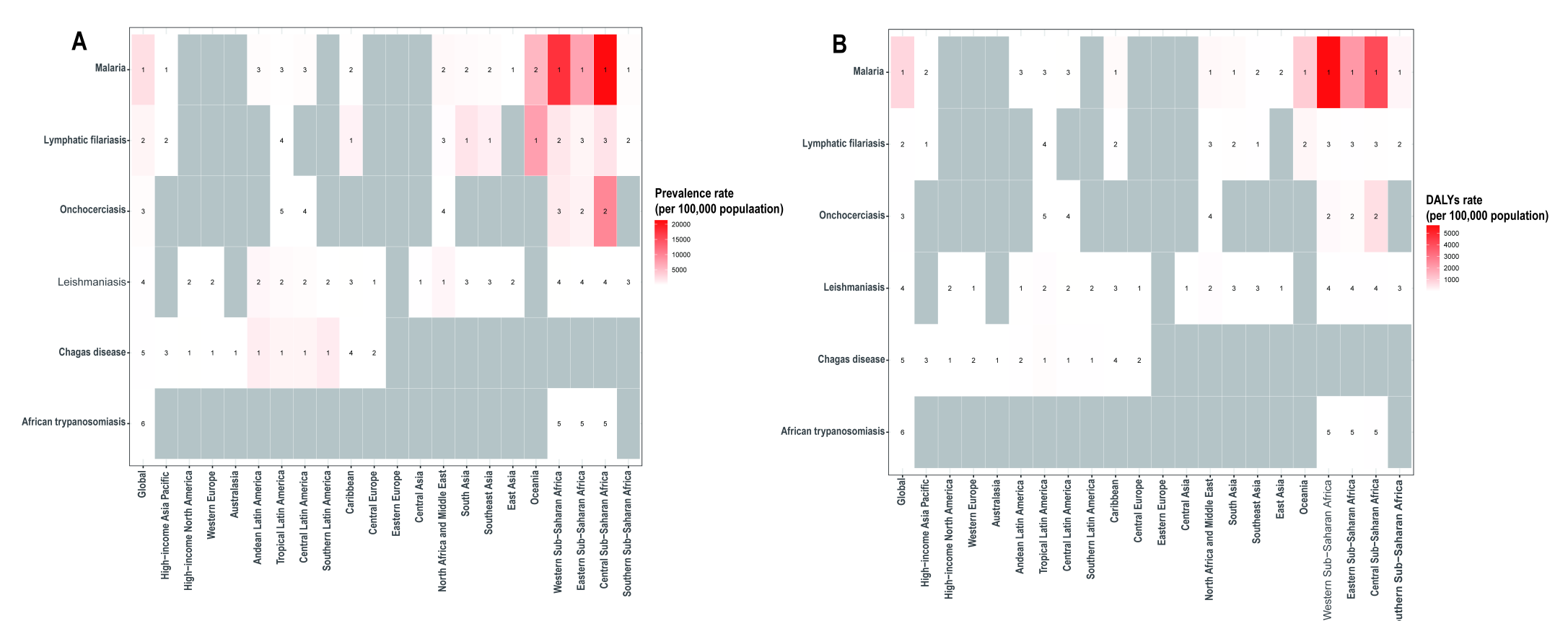


**Figure S1. Vb-pIDP-related burden globally and in different geographic regions in 2021.**

Age-standardized Prevalence rate (**A**) and DALYs (**B**) rate (per 100,000 population) of vb-pIDP.

DALYs: disability-adjusted life years, Vb-pIDP: vector-borne parassitic infectious diseases of poverty.
